# Supplementary material for: Isolation of Unstable Isomers of Lucilactaene and Evaluation of Anti-Inflammatory Activity of Secondary Metabolites Produced by the Endophytic Fungus Fusarium sp. QF001 from the Roots of Scutellaria baicalensis
Source: Molecules. 2020 Feb 19;25(4):923. doi: 10.3390/molecules25040923 (PMC7070625; doi:10.3390/molecules25040923)
Supplement: Supplementary file 1 [file molecules-25-00923-s001.pdf]

molecules

MDPI

Supplementary Materials

Isolation of Unstable Isomers of Lucilactaene and Evaluation of Anti-inflammatory Activity of Secondary Metabolites Produced by an Endophytic Fungus *Fusarium* sp. QF001 from the Roots of *Scutellaria baicalensis*

Sailesh Maharjan[1], Sang Bong Lee[2], Geum Jin Kim[1], Sung Jin Cho[3], Joo-WonNam[1], Jungwook Chin[3,*] and HyukjaeChoi[1,*]

[1] College of Pharmacy, Yeungnam University, Gyeongsan, South Korea; msaileshmaharjan@gmail.com (S.M.); kimgeumjin@naver.com (G.J.K.); jwnam@yu.ac.kr (J.W.N.)
[2] Korea Institute of Medical Microrobotics, Gwangju, 61011, South Korea; sangbongyi1@kimiro.re.kr (S.B.L.)
[3] New Drug Development Center, Daegu-Gyeongbuk Medical Innovation Foundation, Daegu 41061, South Korea; sjcho@dgmif.re.kr (S.J.C.)
* Correspondence:jwchin@dgmif.re.kr (J.C.); h5choil@yu.ac.kr (H.C.)

List of Figures

Figure S1: Semi-preparative RP HPLC chromatogram of fraction C

Figure S2: Chromatographic profiles of C2 and C6 on analytical RP HPLC

Figure S3: Chromatogram of isolated compounds (C6a-C6c and C2a-C2c)

Figure S4: Time-dependent chromatographic monitoring of C6a-C6c on RP HPLC

Figure S5: Time-dependent chromatographic monitoring of C6a-C6c on NP HPLC

Figure S6: ^{1}H NMR spectrum of **1** (250 MHz, $CDCl_3$)

Figure S7: ^{13}C NMR spectrum of **1** (63 MHz, $CDCl_3$)

Figure S8: HR-FAB-MS spectrum of **2**

Figure S9 : ^{1}H NMR spectrum of **2** (600 MHz, $CDCl_3$)

Figure S10 : ^{13}C NMR spectrum of **2** (150 MHz, $CDCl_3$)

Figure S11: COSYspectrum of **2** (600 MHz, $CDCl_3$)

Figure S12: HMQCspectrum of **2** (600 MHz, $CDCl_3$)

Figure S13: HMBCspectrum of **2** (600 MHz, $CDCl_3$)

Figure S14: NOESY spectrum of **2** (600 MHz, $CDCl_3$)

Figure S15: HR-FAB-MS spectrum of **3**

Figure S16: ^{1}H NMR spectrum of **3** (600 MHz, $CDCl_3$)

Figure S17: ^{13}C NMR spectrum of **3** (150 MHz, $CDCl_3$)

Figure S18: COSYspectrum of **3** (600 MHz, $CDCl_3$)

Figure S19: HMQCspectrum of **3** (600 MHz, $CDCl_3$)

Figure S20: HSQC spectrum of **3** (600 MHz, $CDCl_3$)

Figure S21: HMBCspectrum of **3** (600 MHz, $CDCl_3$)

Figure S22: NOESY spectrum of **3** (600 MHz, $CDCl_3$)
Figure S23: ^{1}H NMR spectrum of **4** (250 MHz, $CDCl_3$)
Figure S24: HMQCspectrum of **4** (250 MHz, $CDCl_3$)
Figure S25: HMBCspectrum of **4** (250 MHz, $CDCl_3$)
Figure S26: ^{1}H NMR spectrum of **5** (250 MHz, $CDCl_3$)
Figure S27: ^{13}C NMR spectrum of **5** (63 MHz, $CDCl_3$)
Figure S28: ^{1}H NMR spectrum of **6** (600 MHz, $CDCl_3$)
Figure S29: ^{13}C NMR spectrum of **6** (150 MHz, $CDCl_3$)
Figure S30: NOESY spectra of **6** (600 MHz, $CDCl_3$)
Figure S31: ^{1}H NMR spectrum of **7** (250 MHz, $CDCl_3$)
Figure S32: ^{1}H NMR spectrum of **8** (250 MHz, $CDCl_3$)
Figure S33: ^{13}C NMR spectrum of **8** (63 MHz, $CDCl_3$)
Figure S34: ^{1}H NMR spectrum of **9** (250 MHz, $CDCl_3$)
Figure S35: ^{13}C NMR spectrum of **9** (63 MHz, $CDCl_3$)
Figure S36: Effect on cell viability of compounds **1**-**9**. Cell proliferation in macrophage cell line (RAW264.7) with or without compounds **1**-**9**

List of Tables

Table S1: NMR spectroscopic data (600/ 150 MHz) of **2** in $CDCl_3$
Table S2: NMR spectroscopic data (600/ 150 MHz) of **3** in $CDCl_3$
Table S3: Energy minimization of 13*S**, 14*S**, 15*S** furanopyrrolidone (Case I)
Table S4: Energy minimization of three conformers of 13*S**, 14*S**, 15*R** furanopyrrolidone (Case II)
Table S5: Energy minimization of 13*S**, 14*R**, 15*R** furanopyrrolidone (Case III)
Table S6: Energy minimization of 13*S**, 14*R**, 15*S** furanopyrrolidone (Case IV)
Table S7: Optical rotation calculation of *R*-solaniol
Table S8: Optical rotation calculation of *S*-solaniol

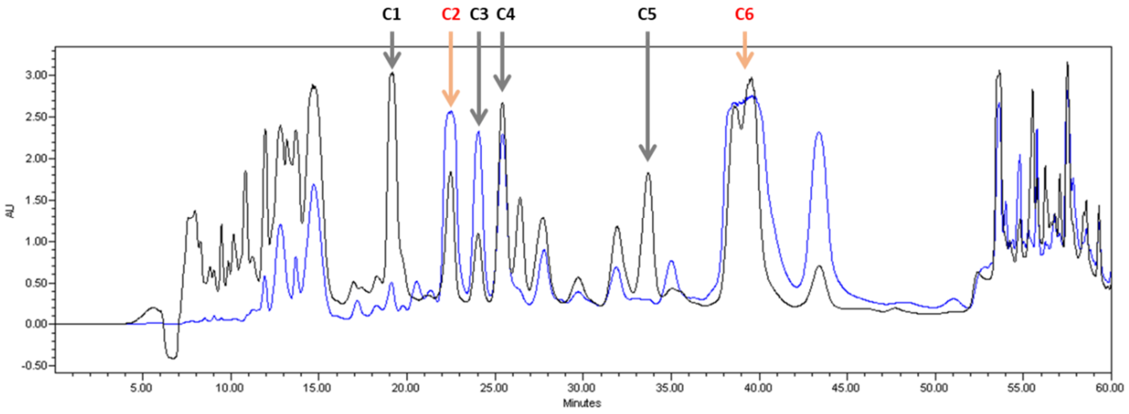

Figure S1: Semi-preparative RP HPLC chromatogram of fraction C [Phenomenex Luna C18, 250 × 10 mm, 5 μm, 2.0 mL/min, mobile phase: A (0.05% formic acid in H_2O), B (0.05% formic acid in acetonitrile), gradient elution: B 44% (0 to 45 min), B 44 to 100% (45 to 50 min), B 100% (50 to 60 min)]

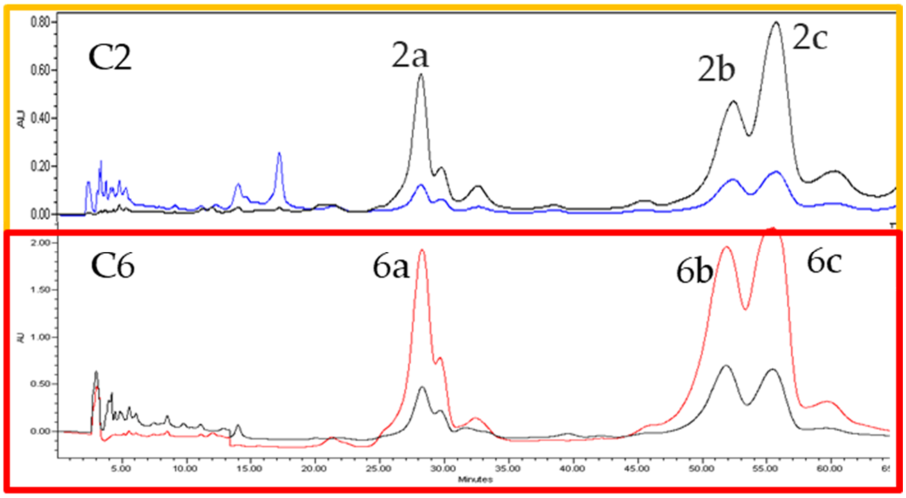

Figure S2: Chromatographic profiles of C2 and C6 on semi-preparative RP HPLC [Phenomenex Luna C18, 250 × 10 mm, 5 μm, 2.0 mL/min, mobile phase: A (0.05% formic acid in H_2O), B (0.05% formic acid in acetonitrile), gradient elution: B 44% (0 to 45 min), B 44 to 100% (45 to 50 min), B 100% (50 to 60 min)]

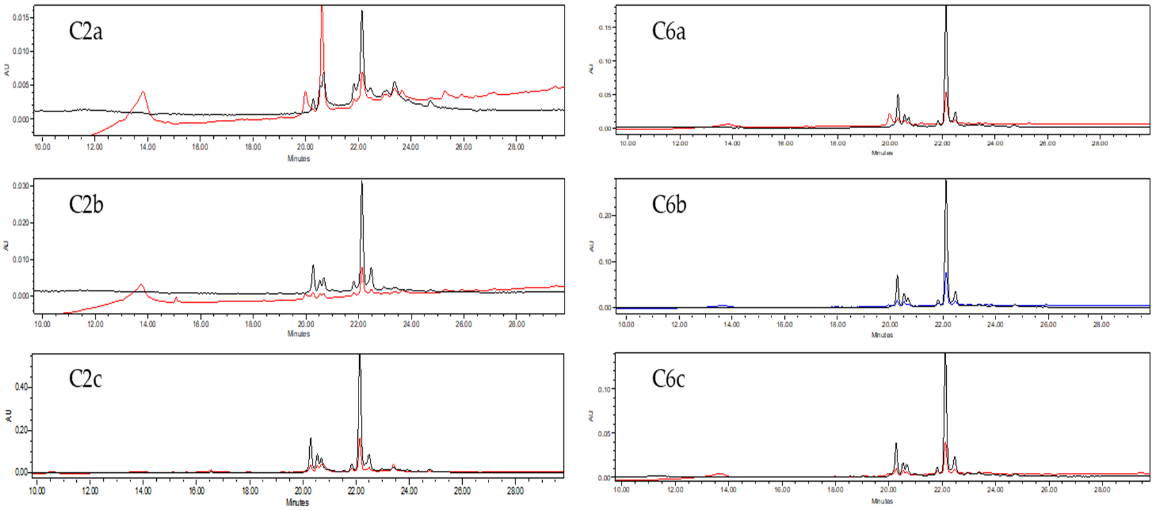

Figure S3: Analytical HPLC chromatogram of isolated compounds (C2a-C2c and C6a-C6c) [Phenomenex Kinetex C18 column, 250 × 4.6 mm, 5 μm, 0.8 mL/min, isocratic elution (H_2O: acetonitrile = 69:31), PDA monitoring at 210 and 365 nm]

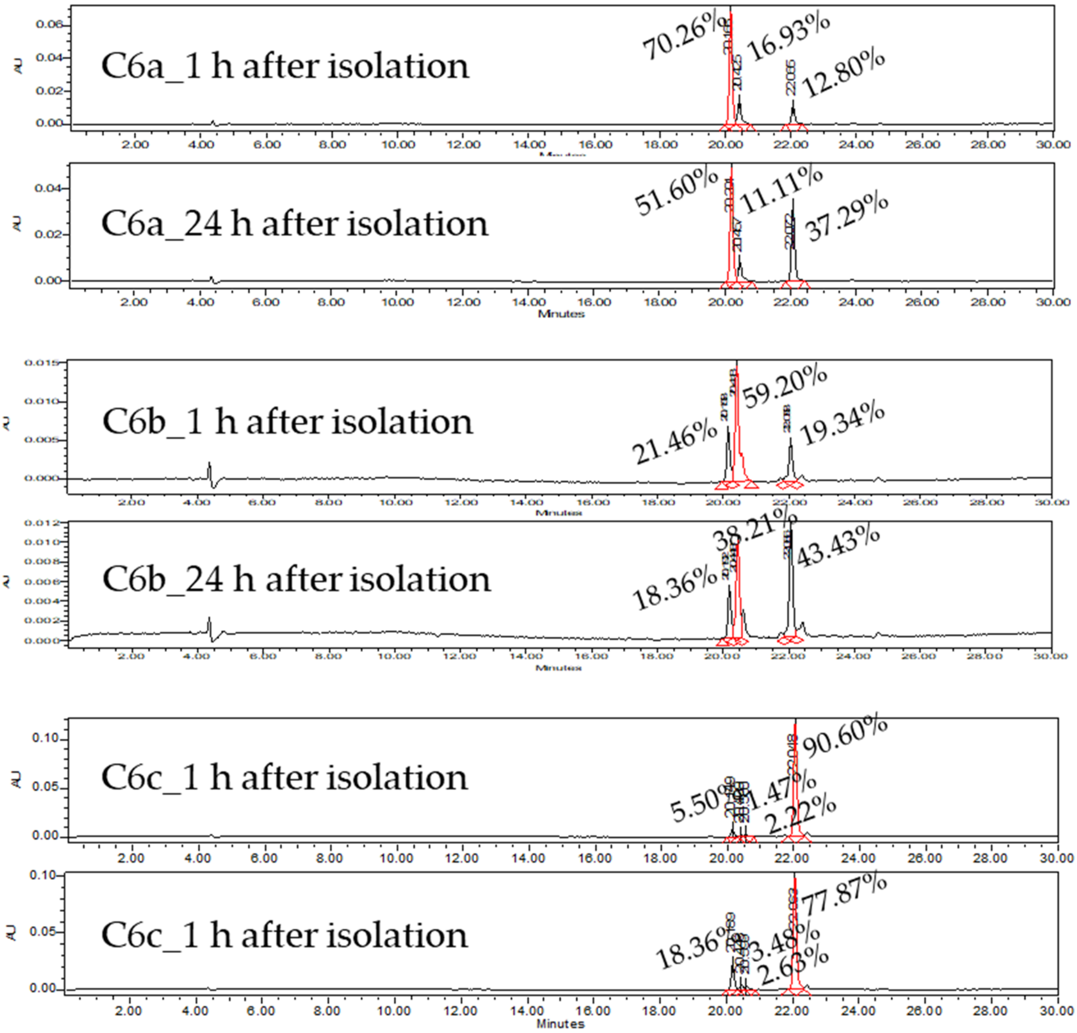

Figure S4: Time-dependent chromatographic monitoring of C6a-C6c on RP HPLC [Phenomenex Kinetex C18 column, 250 × 4.6 mm, 5 µm, 0.8 mL/min, isocratic elution (H_2O:acetonitrile = 69:31), PDA monitoring at 210 and 365 nm]

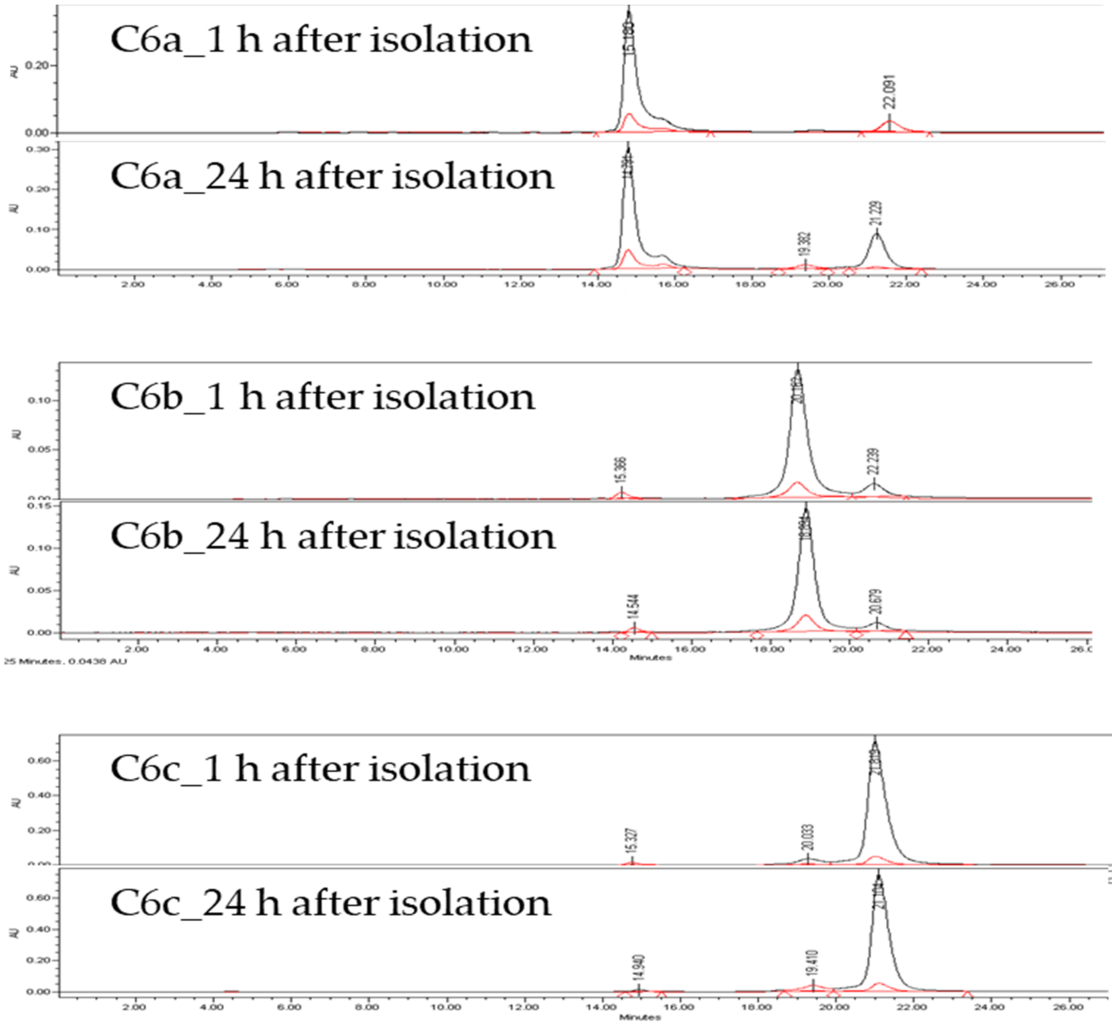

Figure S5: Time-dependent chromatographic monitoring of C6a-C6c on NP HPLC [Inertsil Sil column, 250 × 4.6 mm, 5 μm, 0.7 mL/min, 1-hexane:ethyl acetate = 40:60), PDA monitoring at 210 and 365 nm]

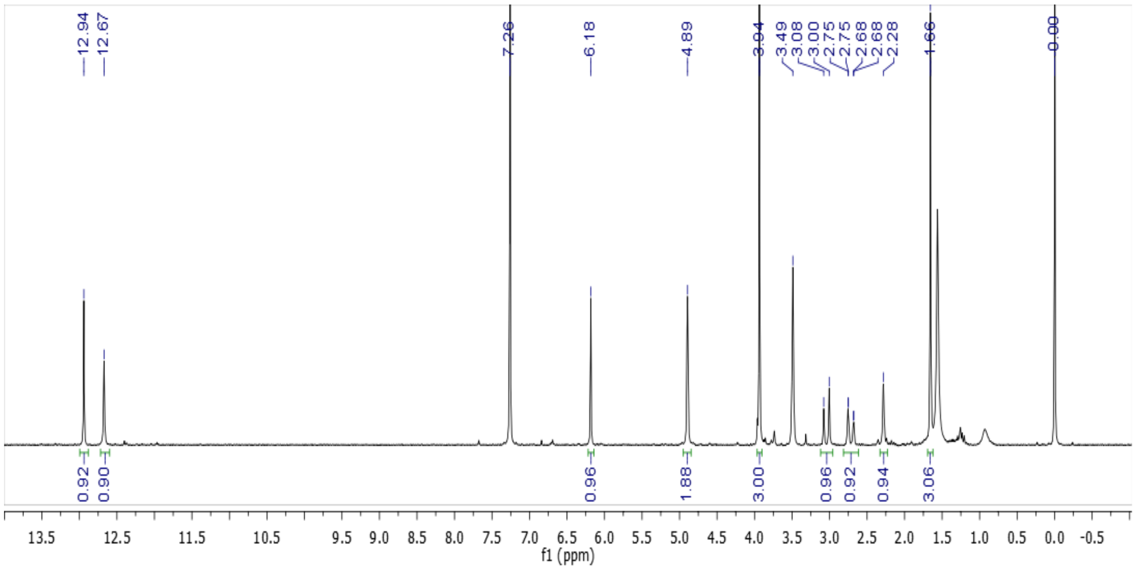

Figure S6: ^{1}H NMR spectrum of **1** (250 MHz, $CDCl_3$)

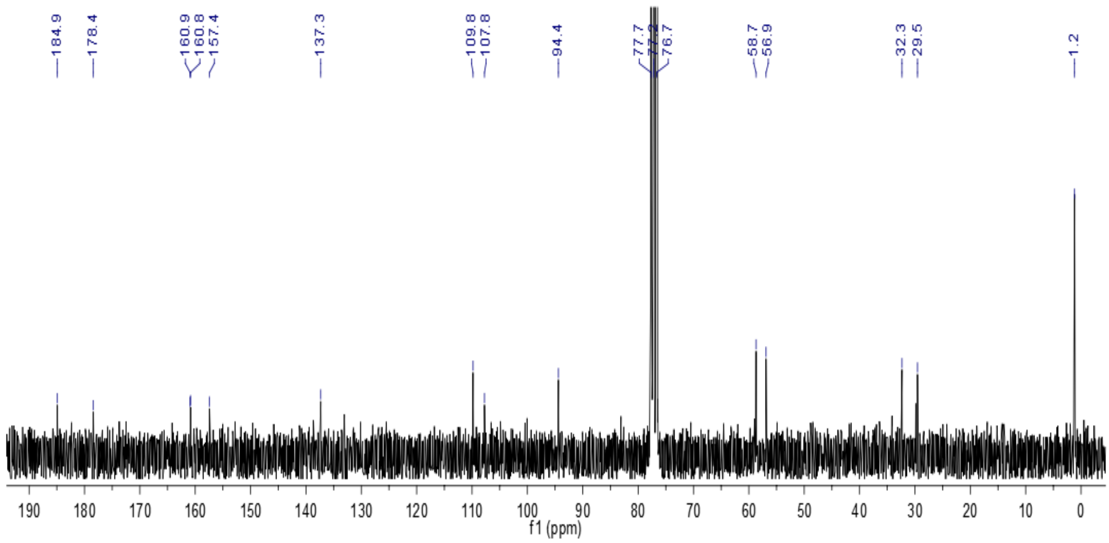

Figure S7: ^{13}C NMR spectrum of **1** (63 MHz, $CDCl_3$)

```
[ Elemental Composition ]
Data   : FAB-R082                          Date : 18-Jul-2018 17:09
Sample: QFIC(NP)_Pk2
Note : m-NBA
Inlet : Direct                             Ion Mode : FAB+
RT : 4.52 min                              Scan#: (200,219)
Elements : C 100/0, H 100/0, N 10/0, O 10/0
Mass Tolerance        : 20ppm, 5mmu if m/z < 250, 10mmu if m/z > 500
Unsaturation (U.S.)  : -0.5 - 50.0

Observed m/z  Int%    Err[ppm / mmu]     U.S.   Composition
  402.1921    100.0     -12.3 /  -4.9    18.5   C 28 H 24 N 3
                        +19.0 /  +7.6    19.0   C 27 H 22 N 4
                        -15.6 /  -6.3    18.0   C 30 H 26 O
                        +15.6 /  +6.3    18.5   C 29 H 24 N O
                         +1.0 /  +0.4    15.0   C 21 H 22 N 8 O
                         -2.3 /  -0.9    14.5   C 23 H 24 N 5 O 2
                         -5.6 /  -2.3    14.0   C 25 H 26 N 2 O 3
                        +11.0 /  +4.4    11.0   C 16 H 22 N 10 O 3
                         +7.7 /  +3.1    10.5   C 18 H 24 N 7 O 4
                         +4.4 /  +1.8    10.0   C 20 H 26 N 4 O 5
                         +1.0 /  +0.4     9.5   C 22 H 28 N O 6
                        -13.6 /  -5.5     6.0   C 14 H 26 N 8 O 6
                        +17.7 /  +7.1     6.5   C 13 H 24 N 9 O 6
                        -16.9 /  -6.8     5.5   C 16 H 28 N 5 O 7
                        +14.4 /  +5.8     6.0   C 15 H 26 N 6 O 7
                        +11.0 /  +4.4     5.5   C 17 H 28 N 3 O 8
                         -3.6 /  -1.4     2.0   C 9 H 26 N 10 O 8
                         +7.7 /  +3.1     5.0   C 19 H 30 O 9
                         -6.9 /  -2.8     1.5   C 11 H 28 N 7 O 9
                        -10.2 /  -4.1     1.0   C 13 H 30 N 4 O 10


[ Theoretical Ion Distribution ]
Molecular Formula : C22 H28 N O6
                    (m/z   402.1917, MW   402.4674, U.S.  9.5)
Base Peak :    402.1917, Averaged MW :    402.4645(a),    402.4652(w)

    m/z       INT.
  402.1917 100.0000 **************************************************
  403.1949  25.0650 ***************
  404.1975   4.2074 **
  405.2002   0.5302
  406.2027   0.0545
  407.2052   0.0048
  408.2077   0.0004
```

Figure S8: HR-FAB-MS spectrum of **2**

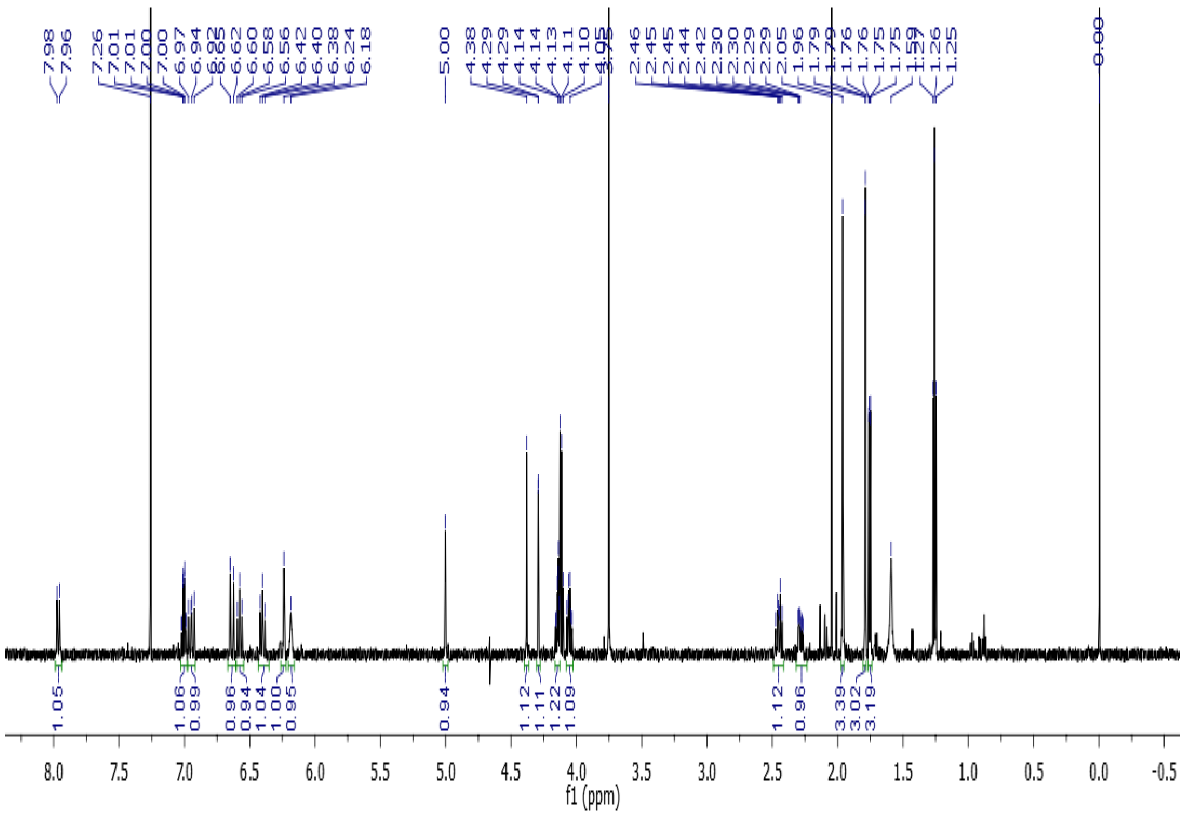

Figure S9: ^{1}H NMR spectrum of **2**(600 MHz, $CDCl_3$)

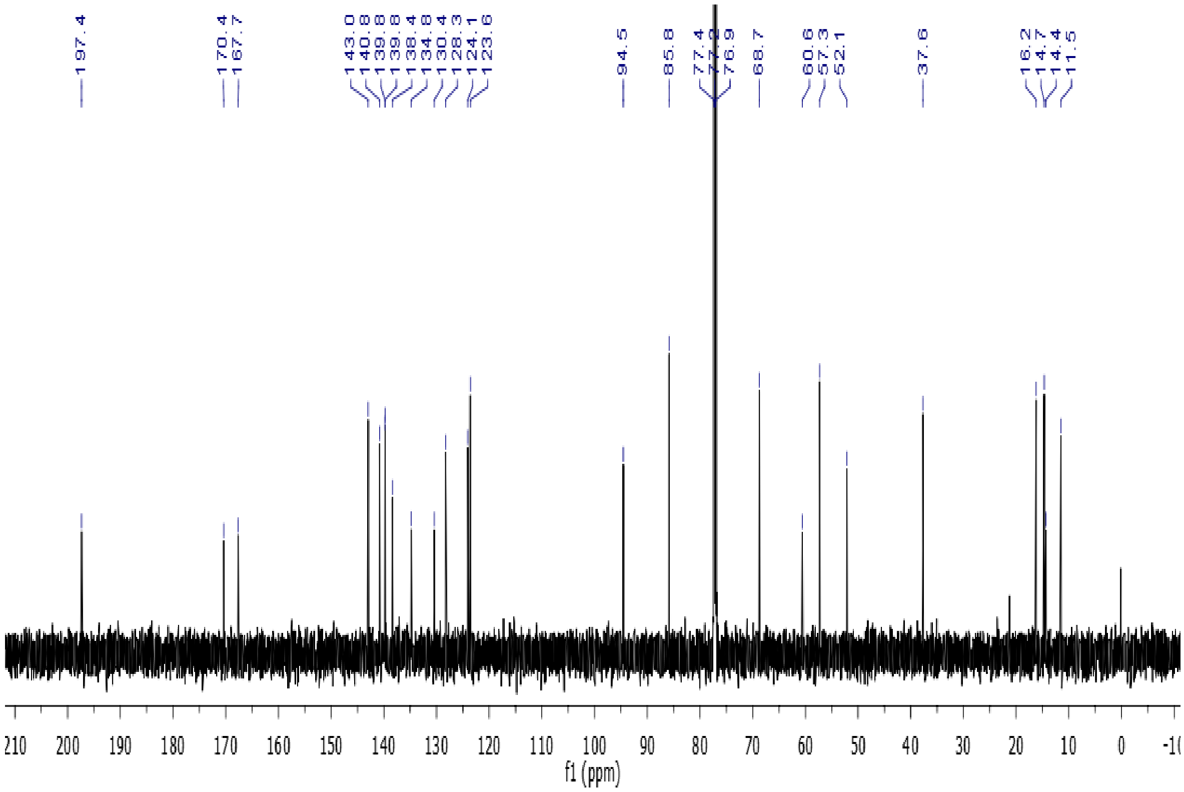

Figure S10: ^{13}C NMR spectrum of **2** (150 MHz, $CDCl_3$)

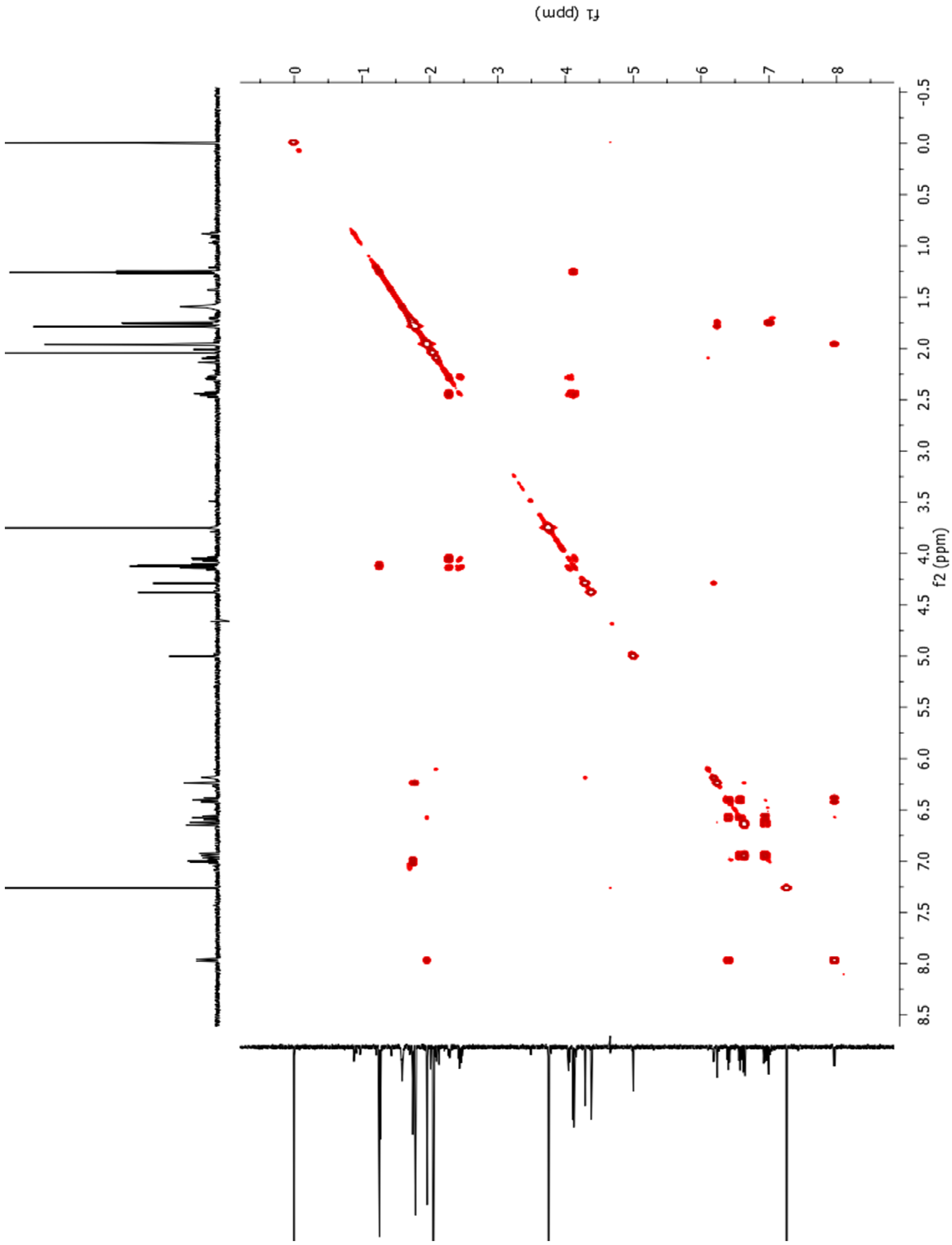

Figure S11: COSY Spectra of **2** (600 MHz, $CDCl_3$)

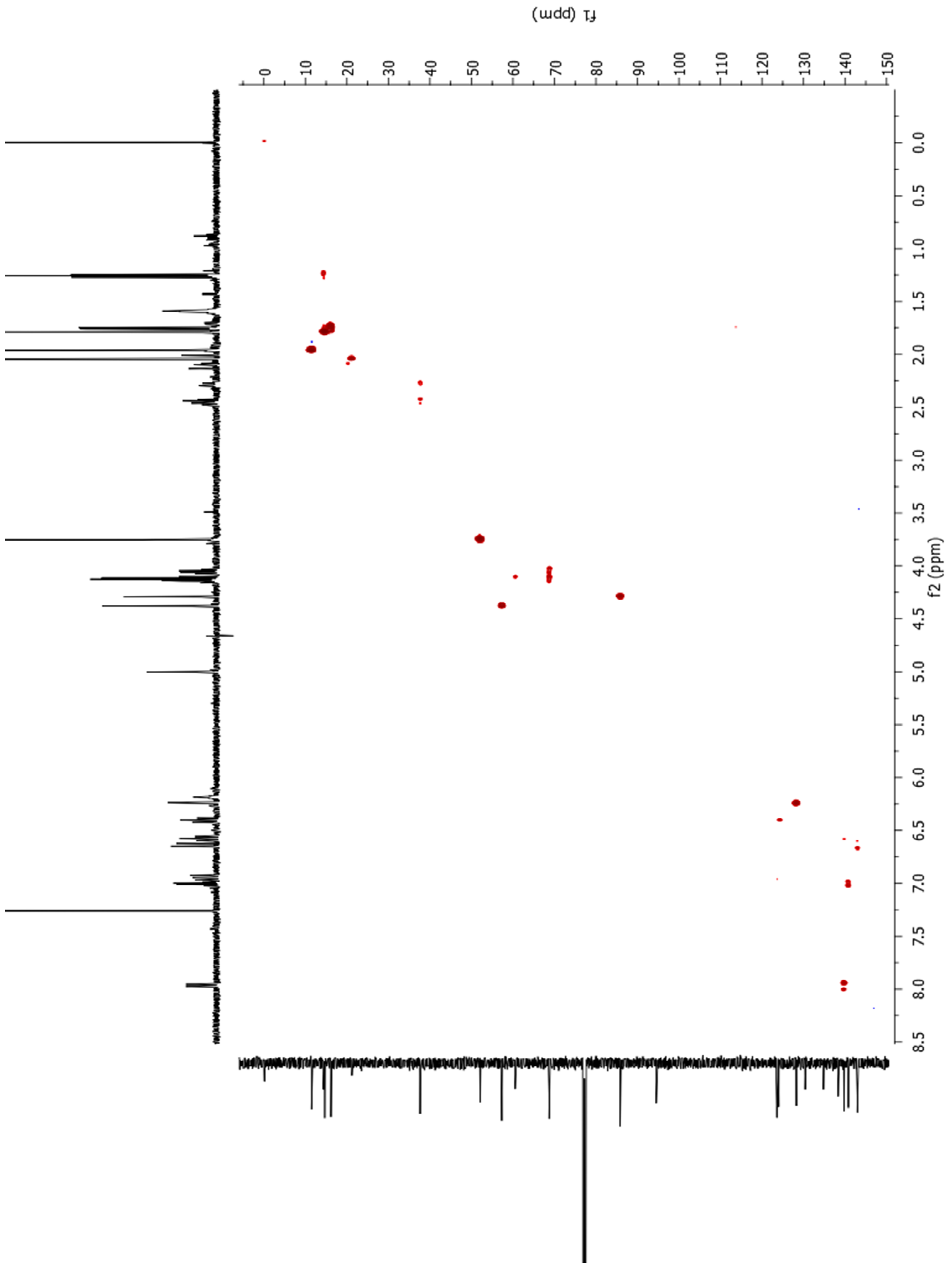

Figure S12: HMQC Spectra of **2** (600 MHz, $CDCl_3$)

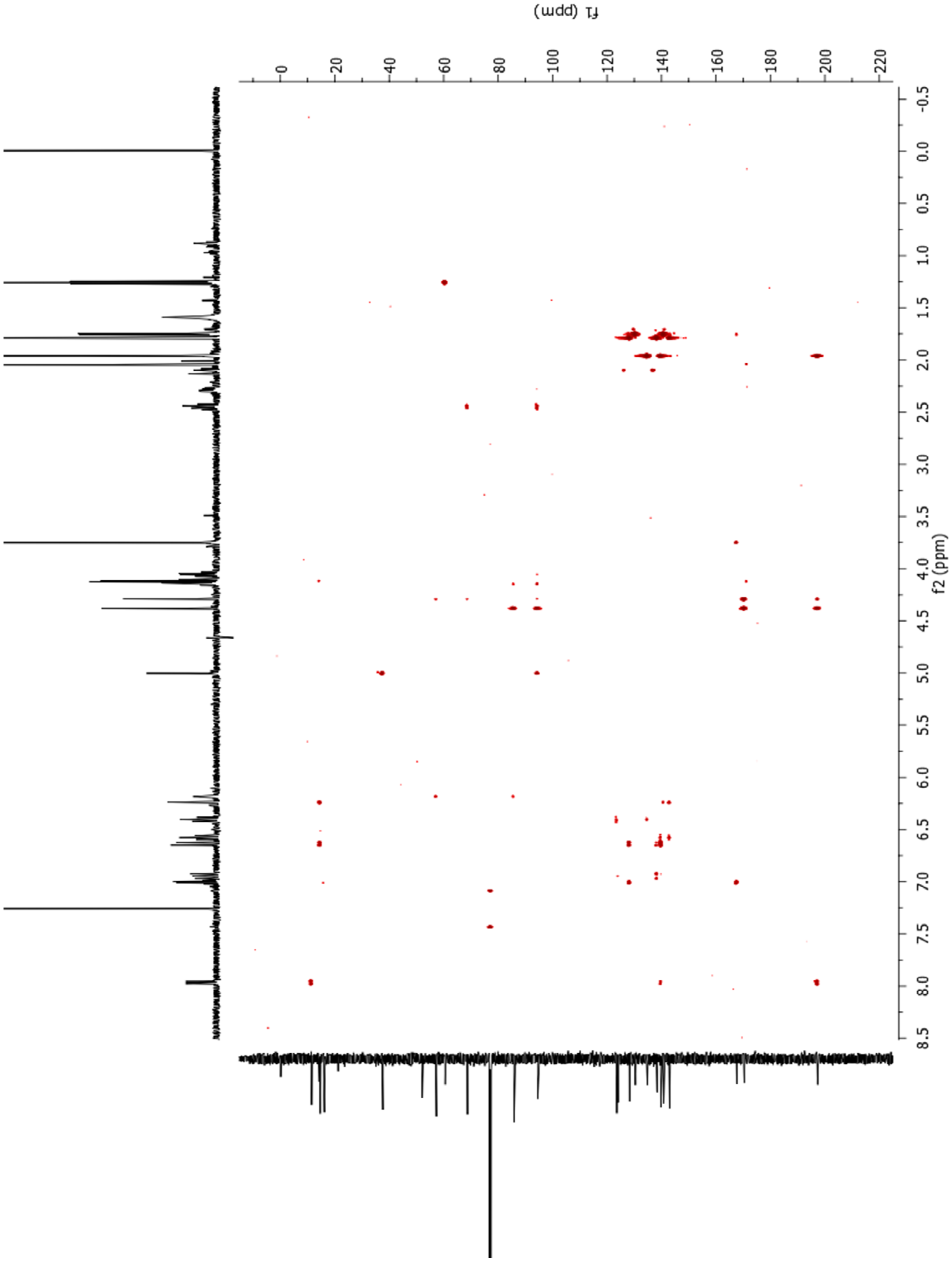

Figure S13: HMBC spectra of **2** (600 MHz, $CDCl_3$)

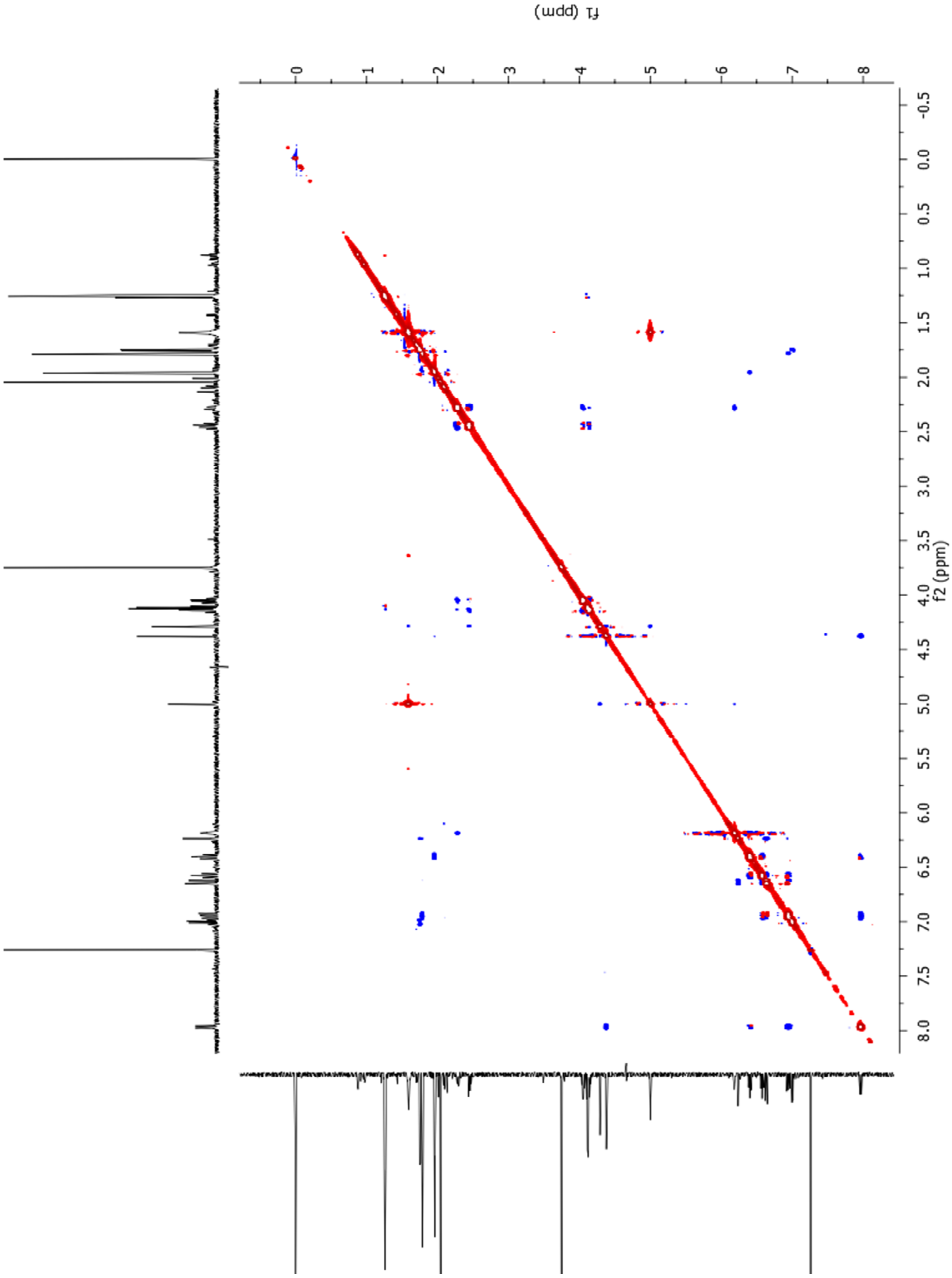

Figure S14: NOESY Spectra of **2** (600 MHz, CDCl3)

[Elemental Composition]
Data : FAB-R083 Date : 18-Jul-2018 17:35
Sample: QFIC(NP)_Pk3
Note : m-NBA
Inlet : Direct Ion Mode : FAB+
RT : 3.80 min Scan#: (172,181)
Elements : C 100/0, H 100/0, N 10/0, O 10/0
Mass Tolerance : 20ppm, 5mmu if m/z < 250, 10mmu if m/z > 500
Unsaturation (U.S.) : -0.5 - 50.0

Observed m/z	Int%	Err[ppm / mmu]	U.S.	Composition
402.1917	28.5	-13.1 / -5.3	18.5	C 28 H 24 N 3
		+18.1 / +7.3	19.0	C 27 H 22 N 4
		-16.5 / -6.6	18.0	C 30 H 26 O
		+14.8 / +6.0	18.5	C 29 H 24 N O
		+0.2 / +0.1	15.0	C 21 H 22 N 8 O
		-3.1 / -1.3	14.5	C 23 H 24 N 5 O 2
		-6.5 / -2.6	14.0	C 25 H 26 N 2 O 3
		+10.2 / +4.1	11.0	C 16 H 22 N 10 O 3
		+6.9 / +2.8	10.5	C 18 H 24 N 7 O 4
		+3.5 / +1.4	10.0	C 20 H 26 N 4 O 5
		+0.2 / +0.1	9.5	C 22 H 28 N O 6
		-14.4 / -5.8	6.0	C 14 H 26 N 8 O 6
		+16.9 / +6.8	6.5	C 13 H 24 N 9 O 6
		-17.7 / -7.1	5.5	C 16 H 28 N 5 O 7
		+13.5 / +5.4	6.0	C 15 H 26 N 6 O 7
		+10.2 / +4.1	5.5	C 17 H 28 N 3 O 8
		-4.4 / -1.8	2.0	C 9 H 26 N 10 O 8
		+6.9 / +2.8	5.0	C 19 H 30 O 9
		-7.7 / -3.1	1.5	C 11 H 28 N 7 O 9
		-11.1 / -4.5	1.0	C 13 H 30 N 4 O 10

[Theoretical Ion Distribution]
Molecular Formula : C22 H28 N O6
(m/z 402.1917, MW 402.4674, U.S. 9.5)
Base Peak : 402.1917, Averaged MW : 402.4645(a), 402.4652(w)

m/z	INT.	
402.1917	100.0000	**
403.1949	25.0650	***************
404.1975	4.2074	**
405.2002	0.5302	
406.2027	0.0545	
407.2052	0.0048	
408.2077	0.0004	

Figure S15: HR-FAB-MS spectrum of **3**

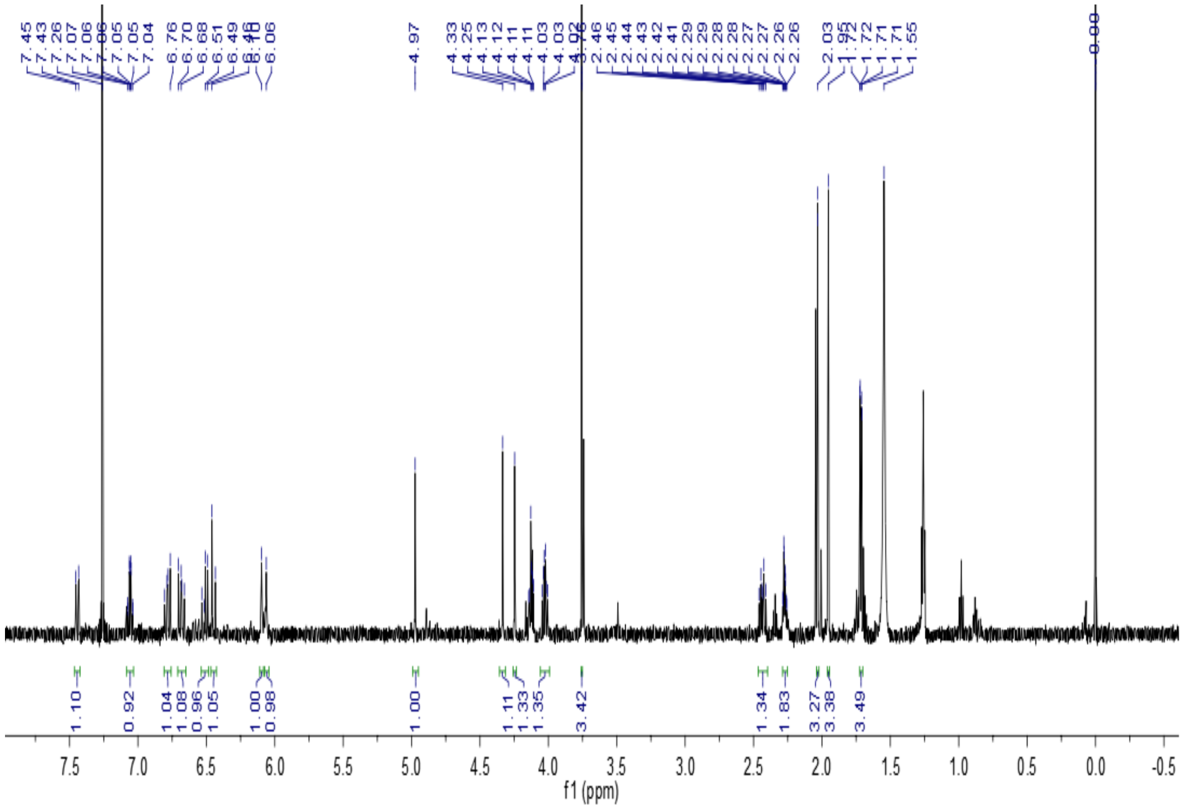

Figure S16: ^{1}H NMR spectrum of **3** (600 MHz, $CDCl_3$)

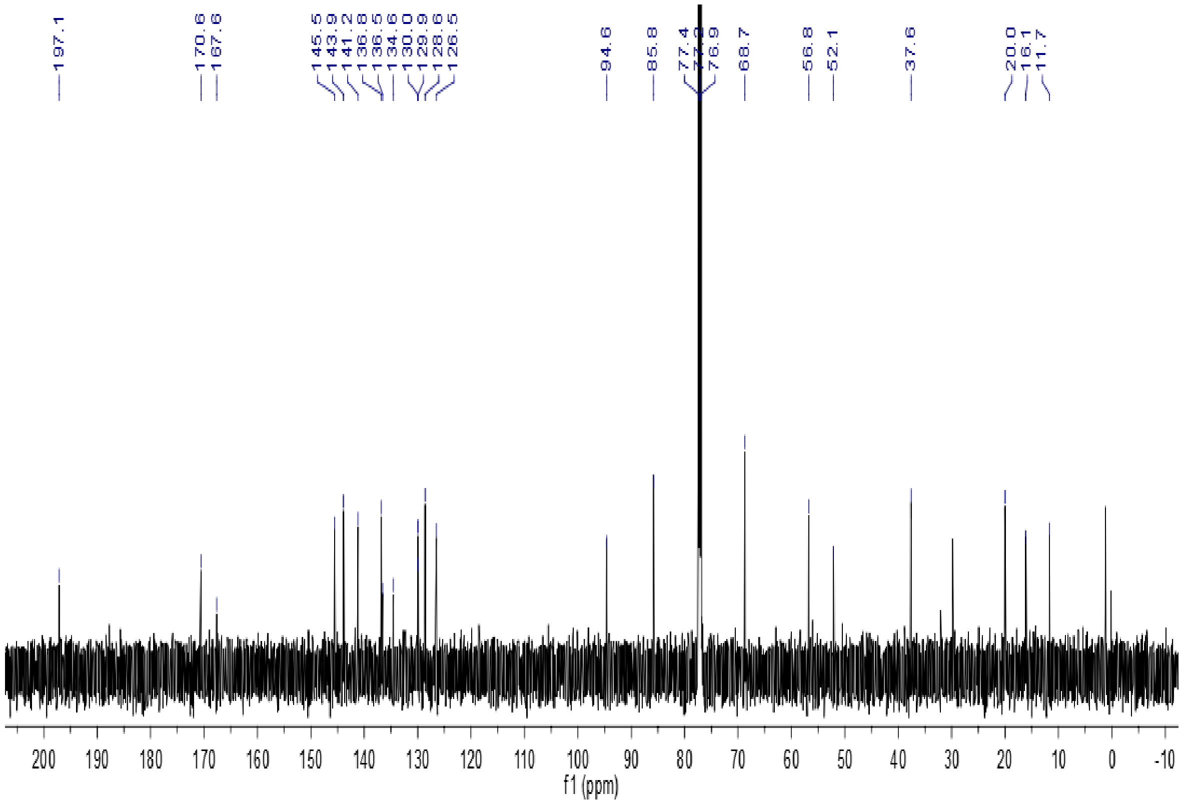

Figure S17: ^{13}C NMR spectrum of **3** (150 MHz, $CDCl_3$)

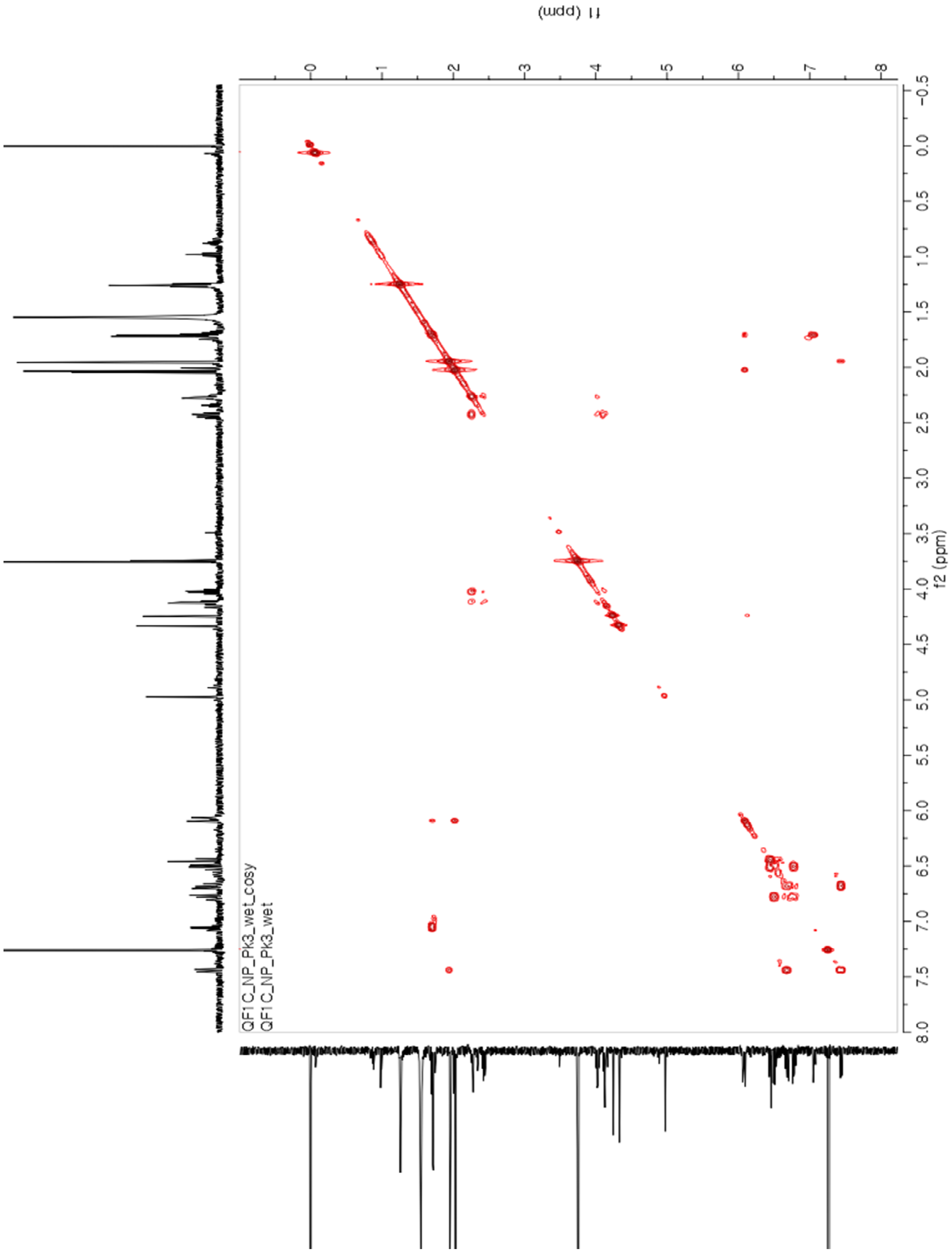

Figure S18: COSY Spectra of **3** (600 MHz, $CDCl_3$)

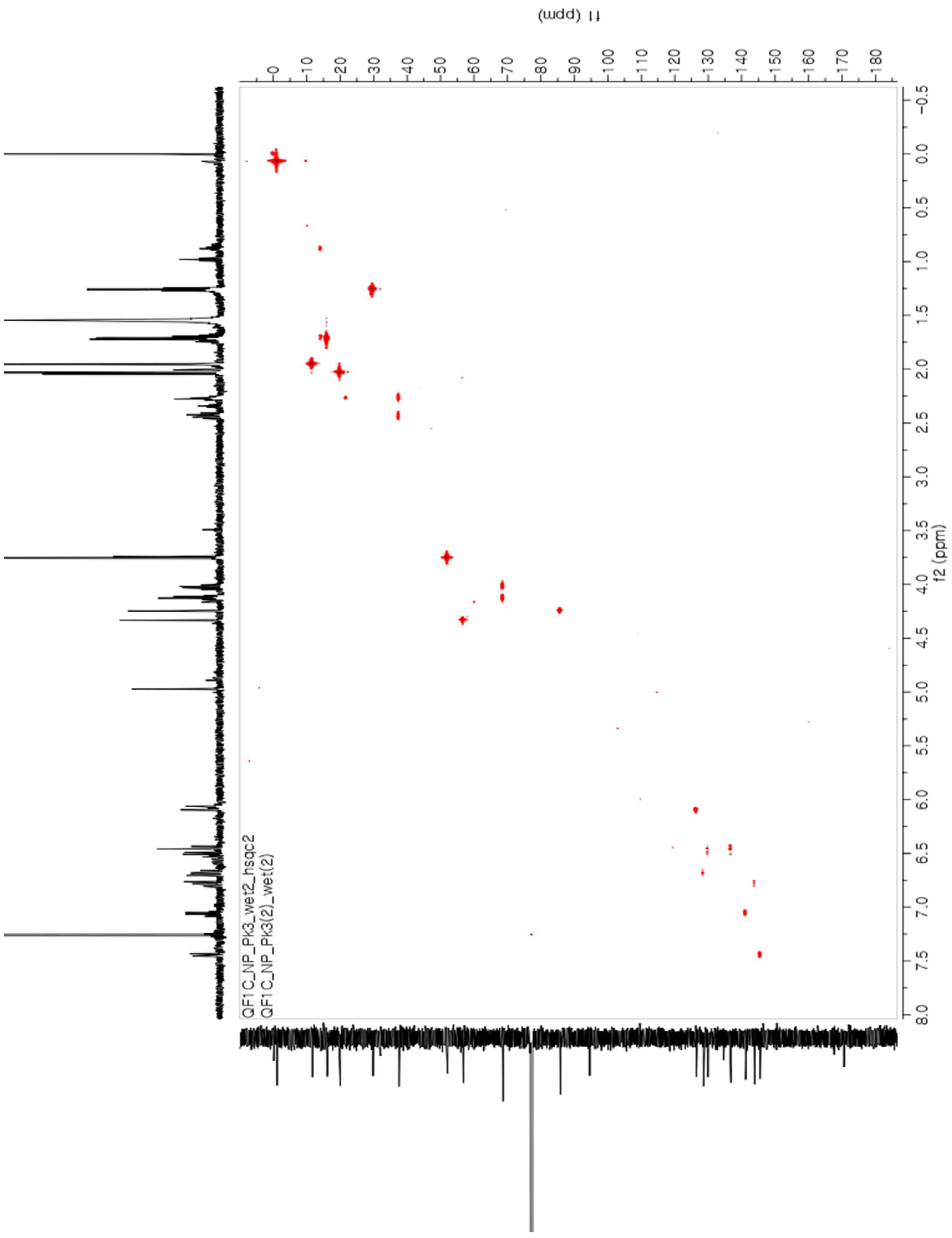

Figure S19: HMQC spectra of **3**(600 MHz, $CDCl_3$)

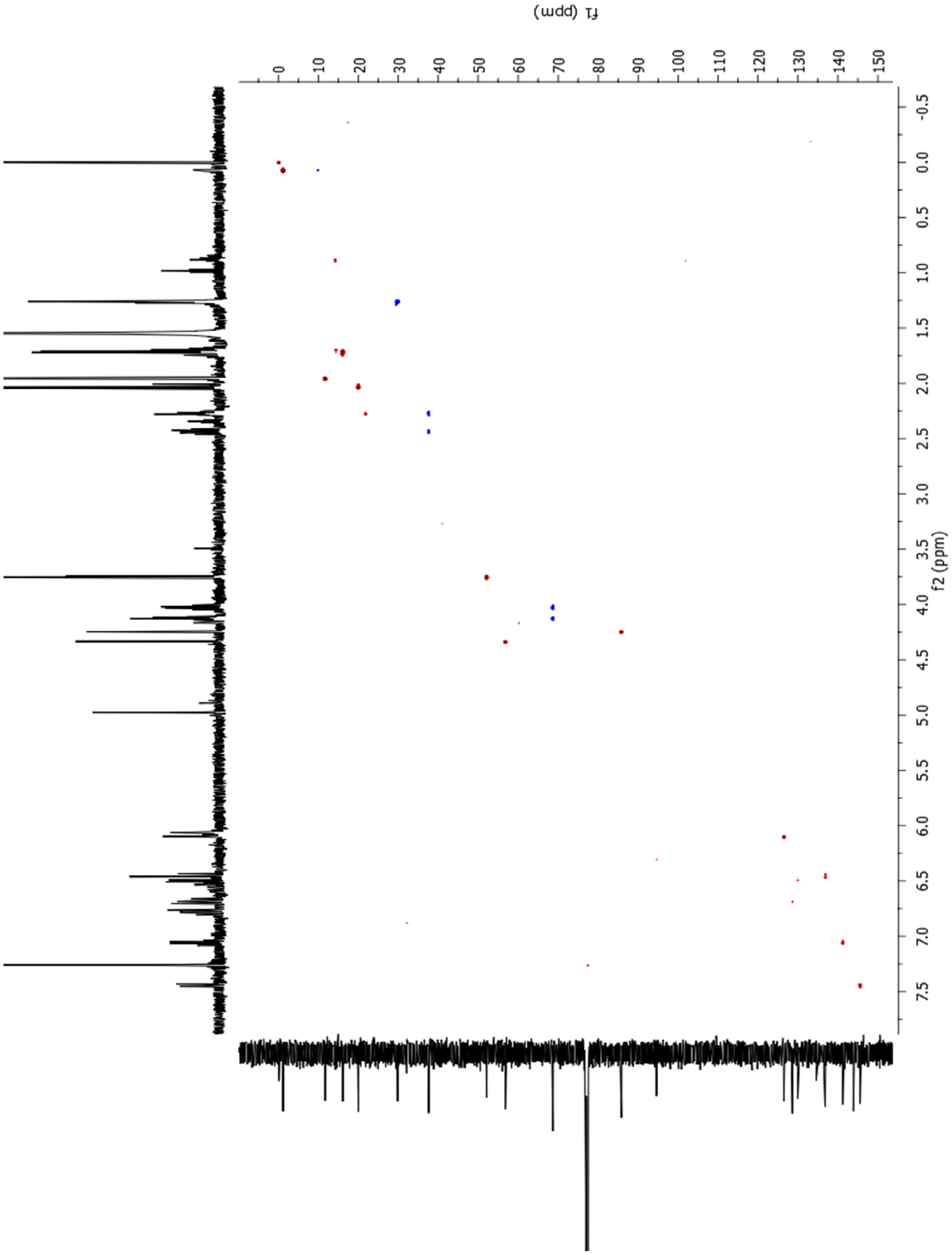

Figure S20: HSQC spectra of **3** (600 MHz, $CDCl_3$)

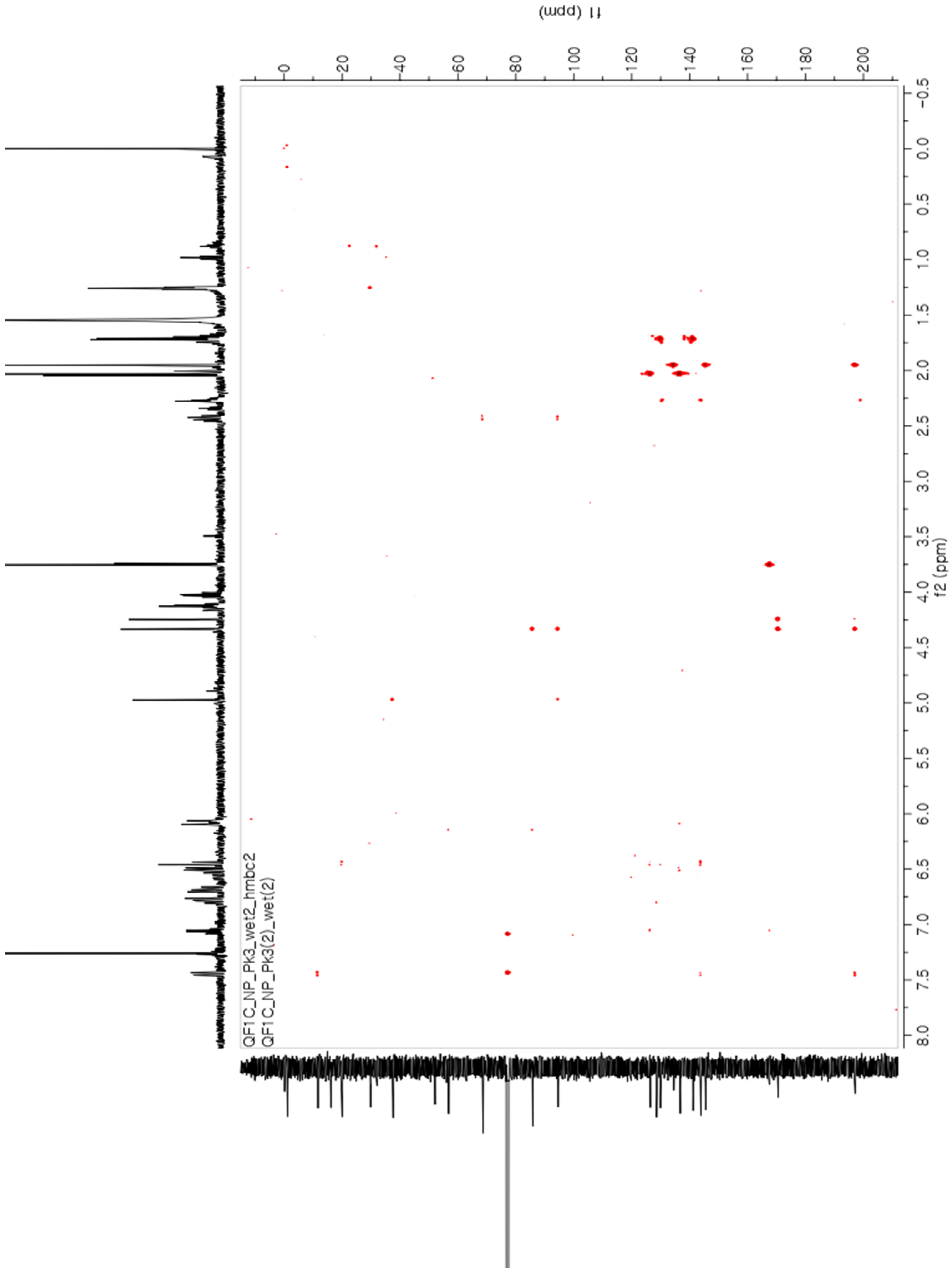

Figure S21: HMBC spectra of **3** (600 MHz, $CDCl_3$)

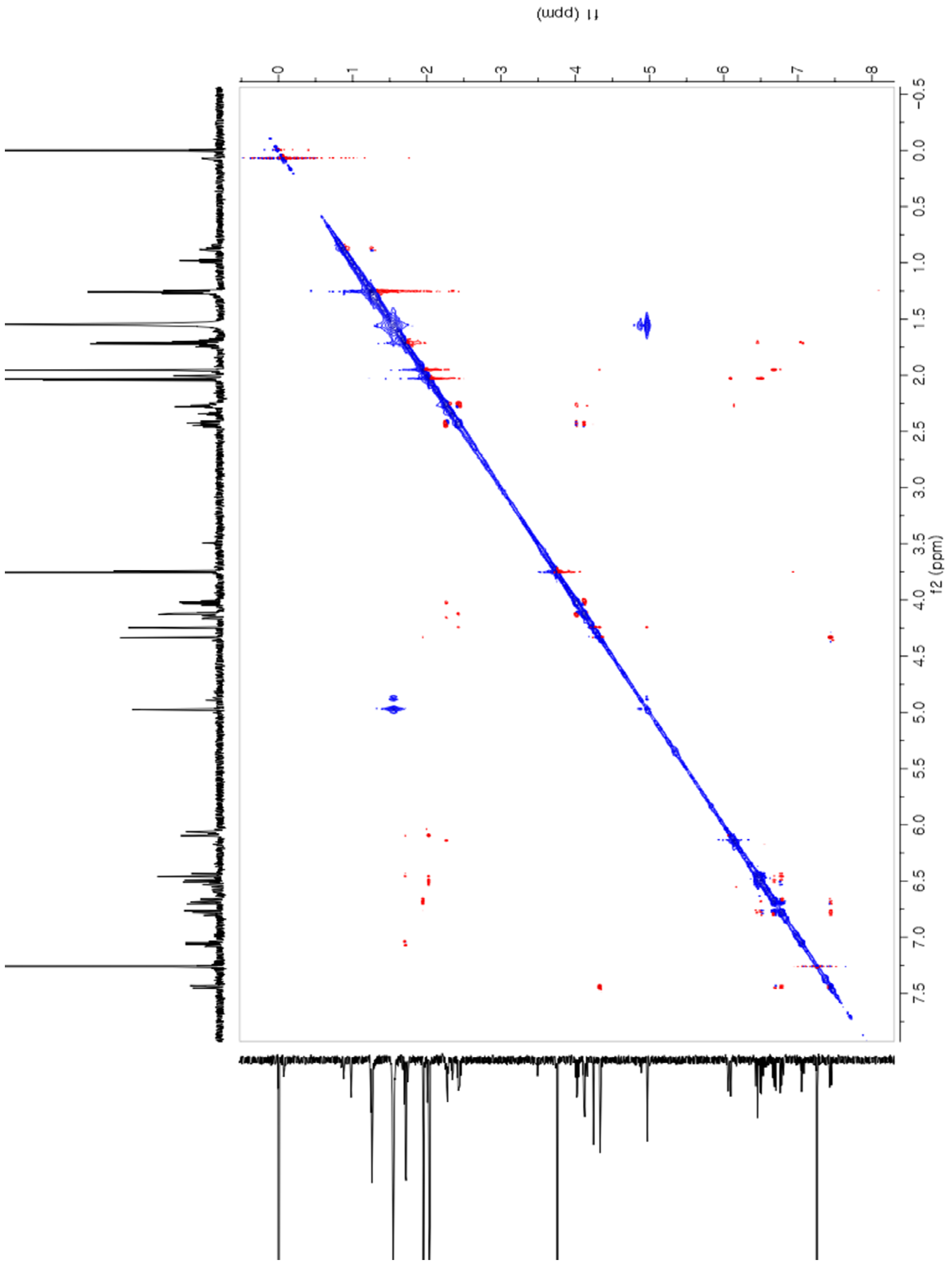

Figure S22: NOESY spectra of **3** (600 MHz, $CDCl_3$)

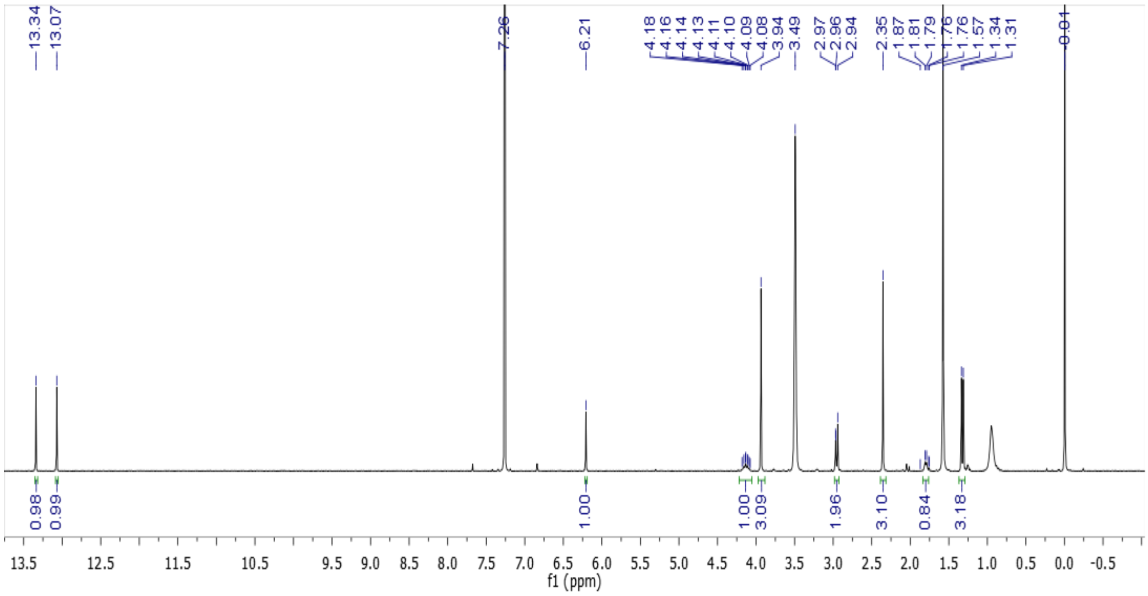

Figure S23: ^{1}H NMR spectrum of **4** (250 MHz, $CDCl_3$)

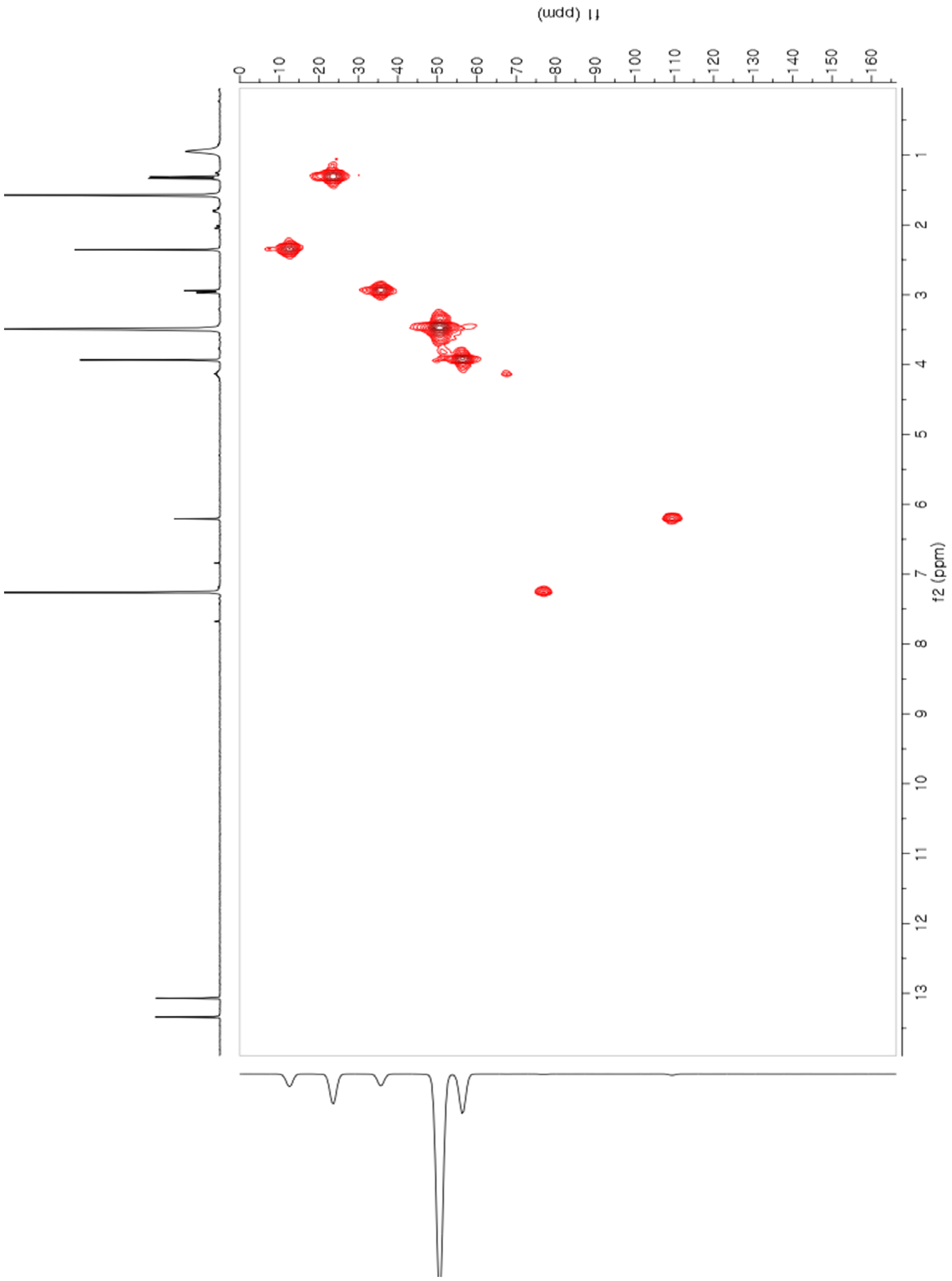

Figure S24: HMQC spectra of **4** (250 MHz, $CDCl_3$)

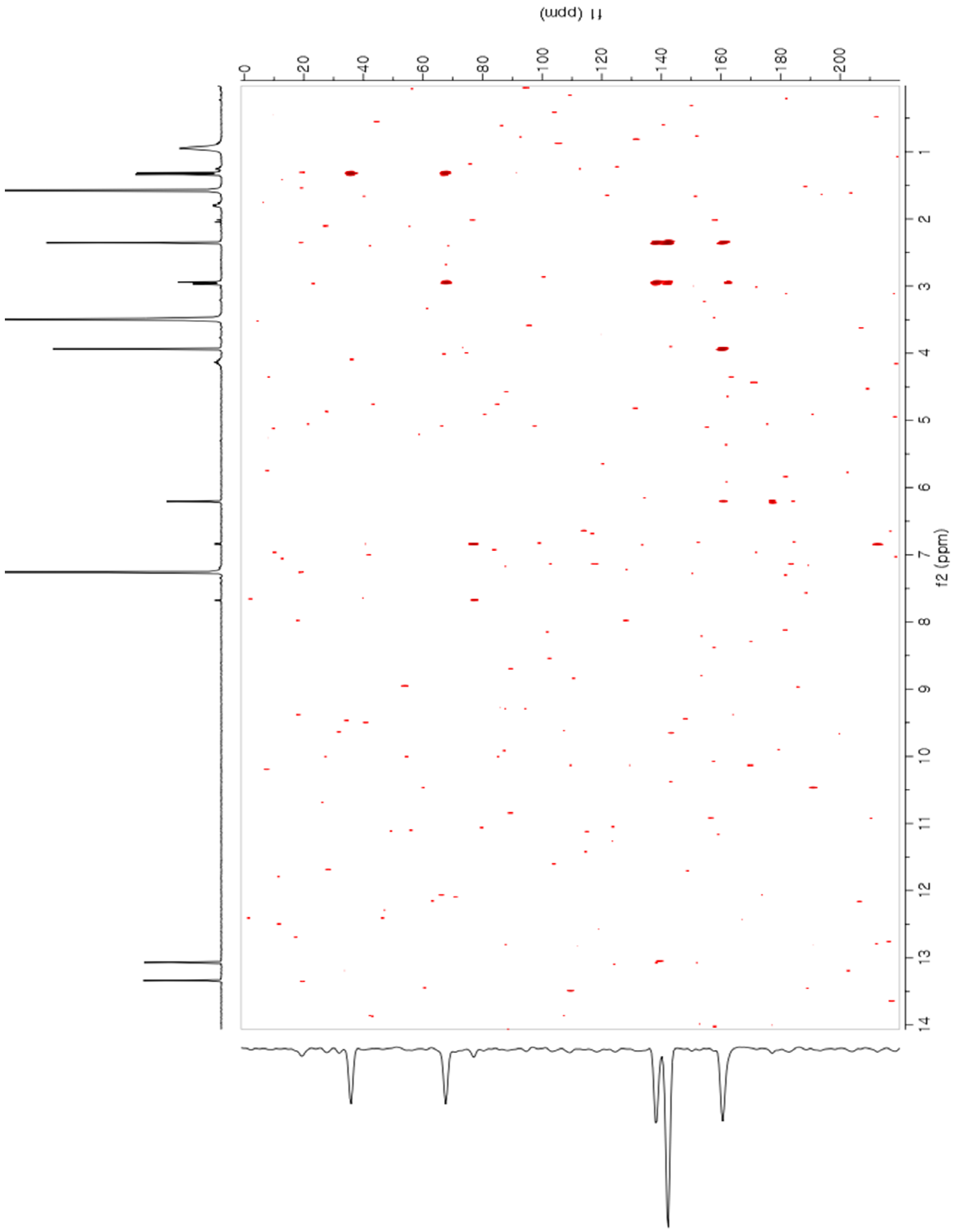

Figure S25: HMBC spectra of **4** (250MHz, $CDCl_3$)

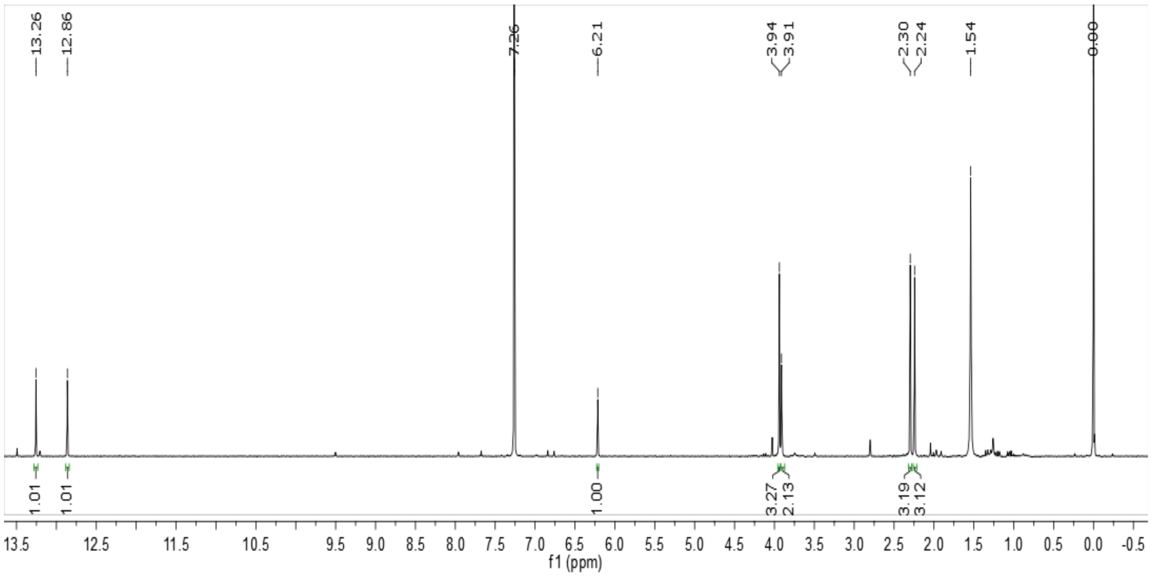

Figure S26: ^{1}H NMR spectrum of **5** (250 MHz, $CDCl_3$)

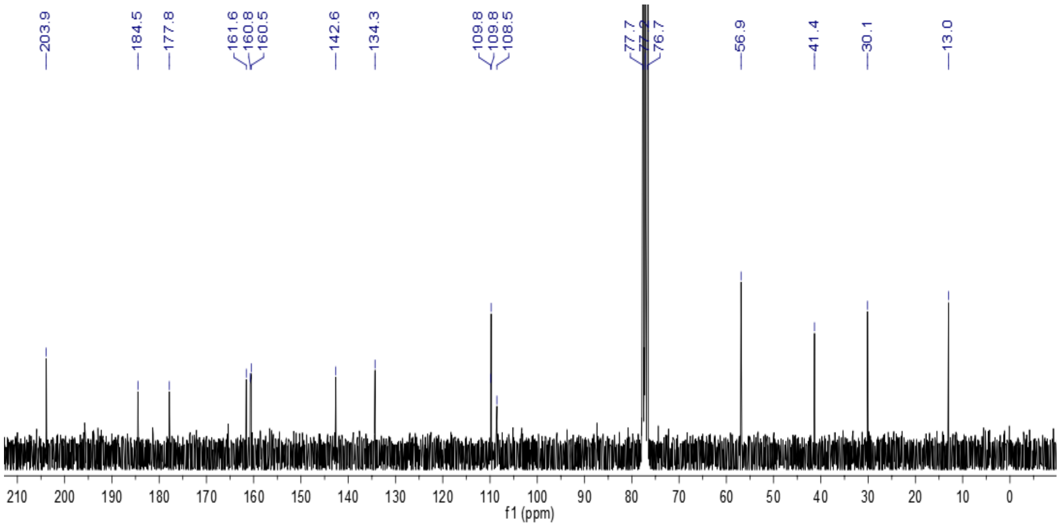

Figure S27: ^{13}C NMR spectrum of **5** (63 MHz, $CDCl_3$)

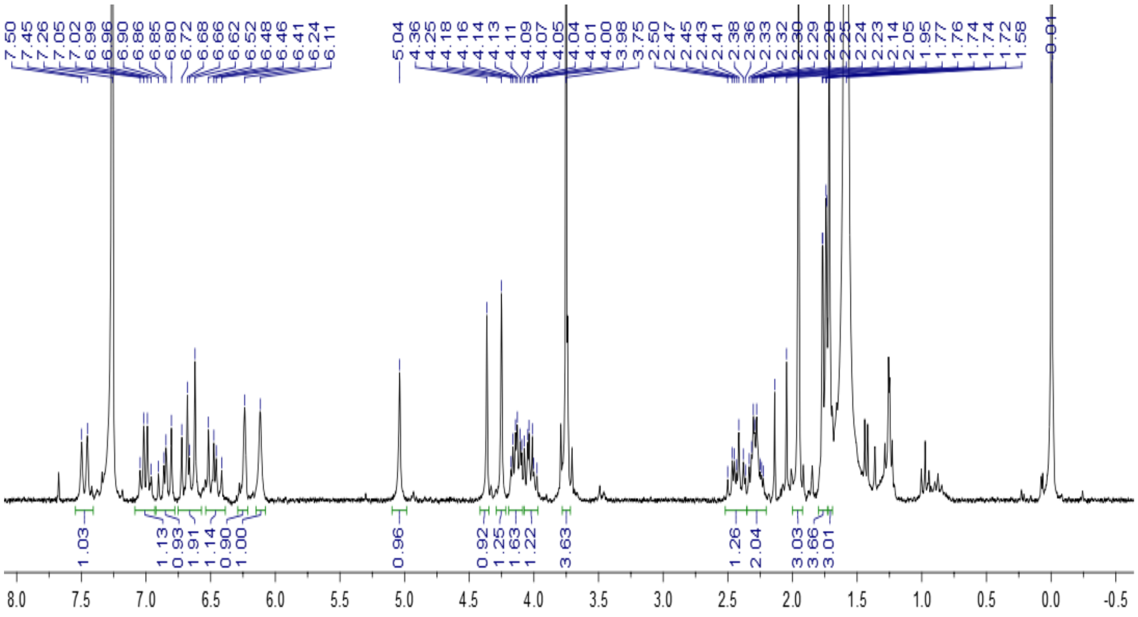

Figure S28: ^{1}H NMR spectrum of **6** (600 MHz, $CDCl_3$)

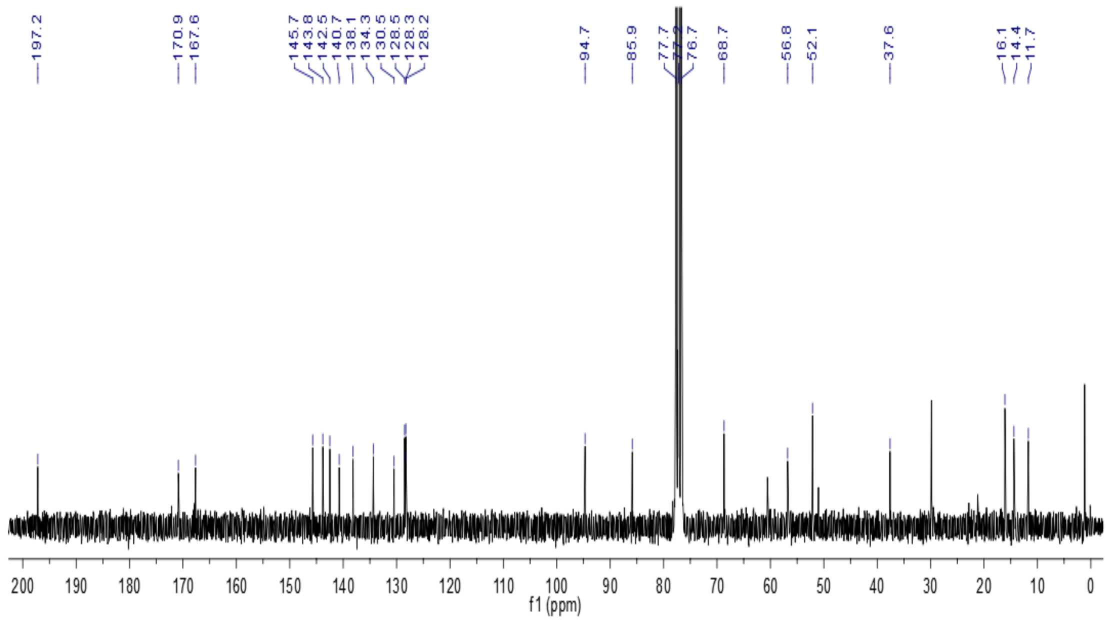

Figure S1: ^{13}C NMR spectrum of **6** (150 MHz, $CDCl_3$)

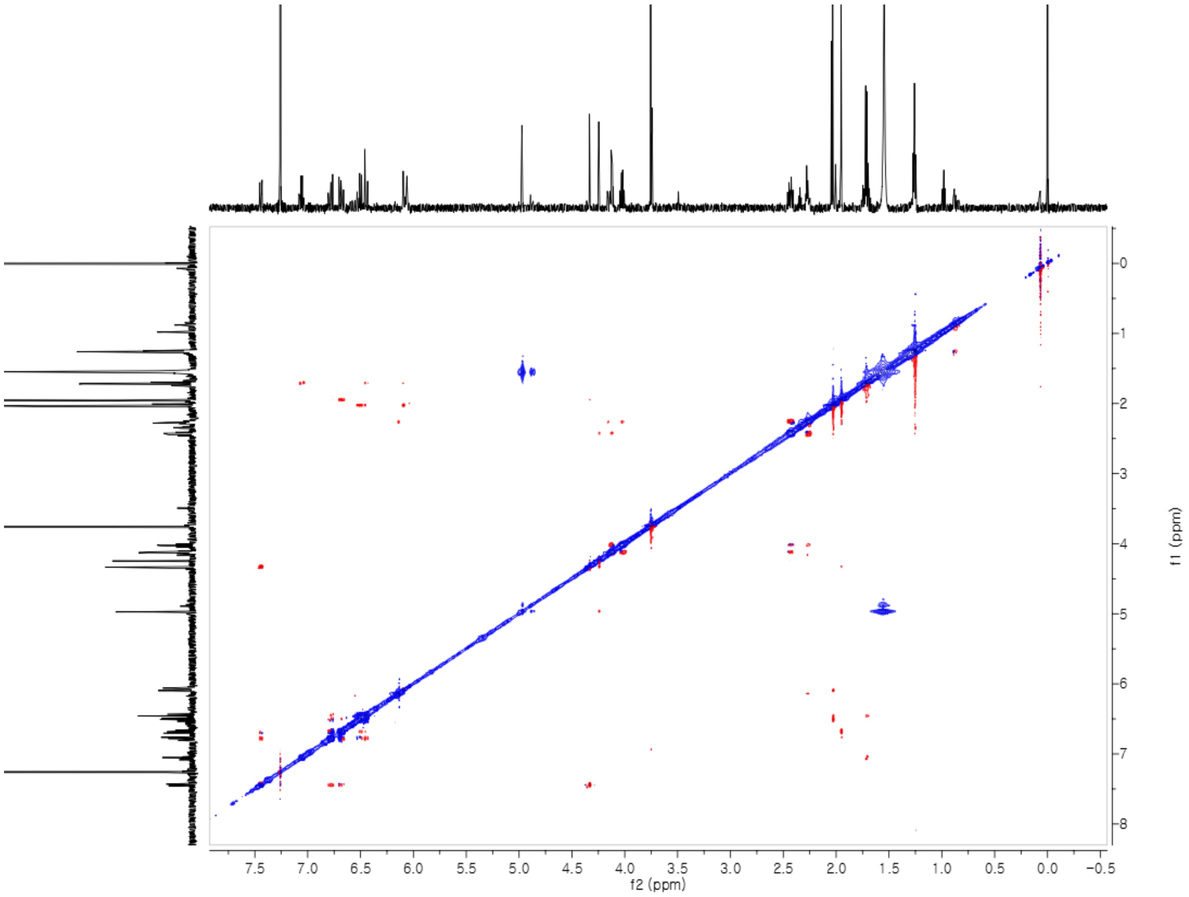

Figure S30: NOESY spectra of **6** (250 MHz, $CDCl_3$)

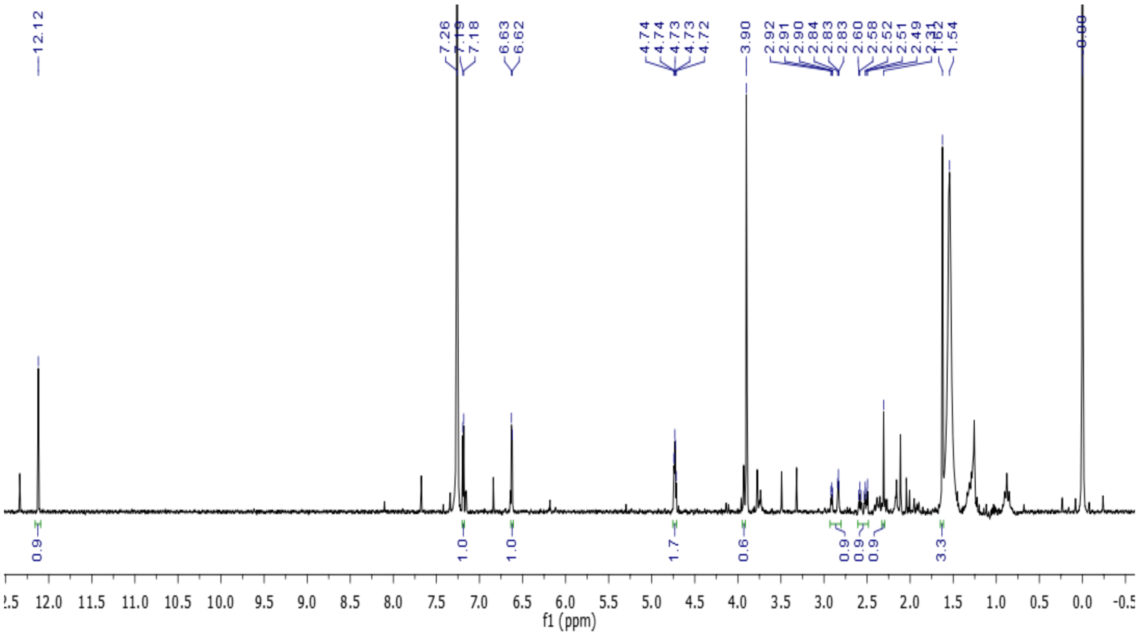

Figure S31: ^1H NMR spectrum of **7** (250 MHz, $CDCl_3$)

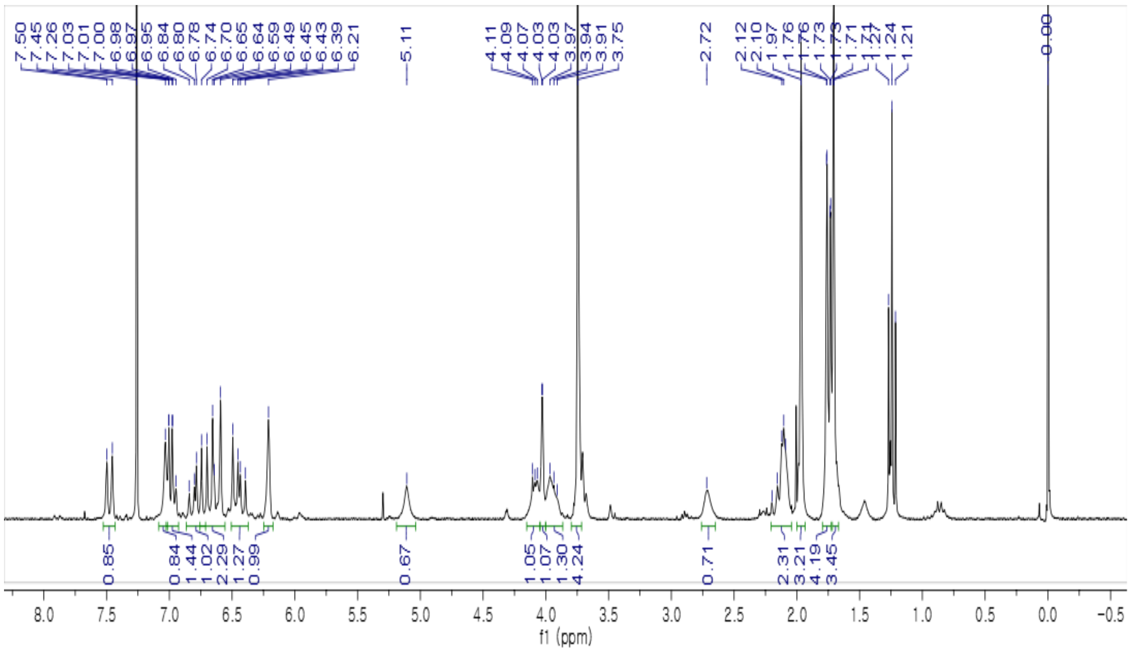

Figure S32: ^{1}H NMR spectrum of **8** (250 MHz, $CDCl_3$)

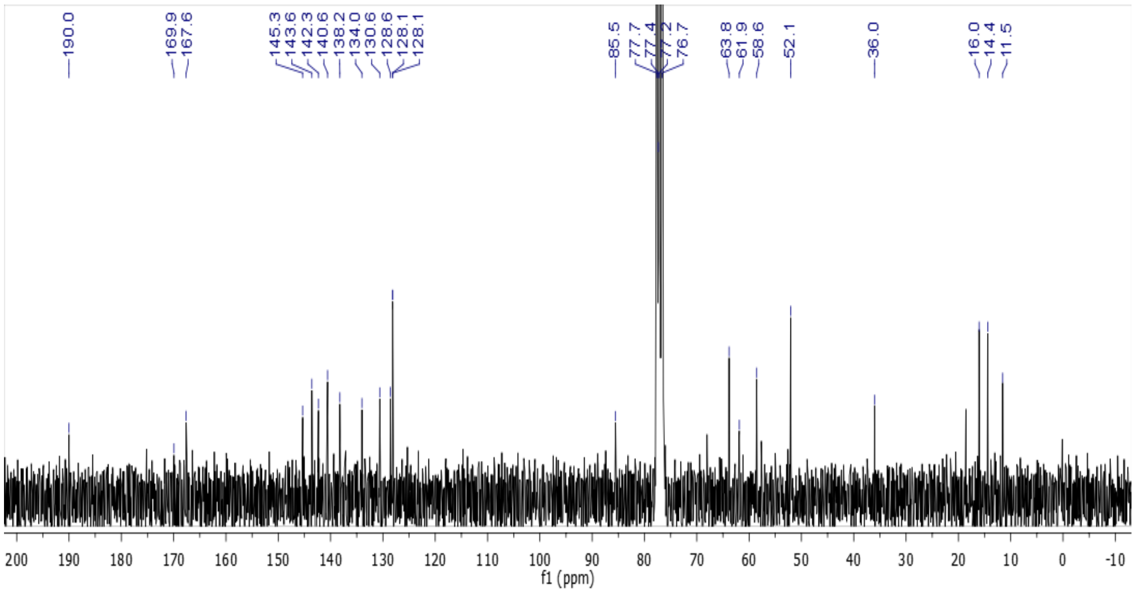

Figure S33: ^{13}C NMR spectrum of **8** (63 MHz, $CDCl_3$)

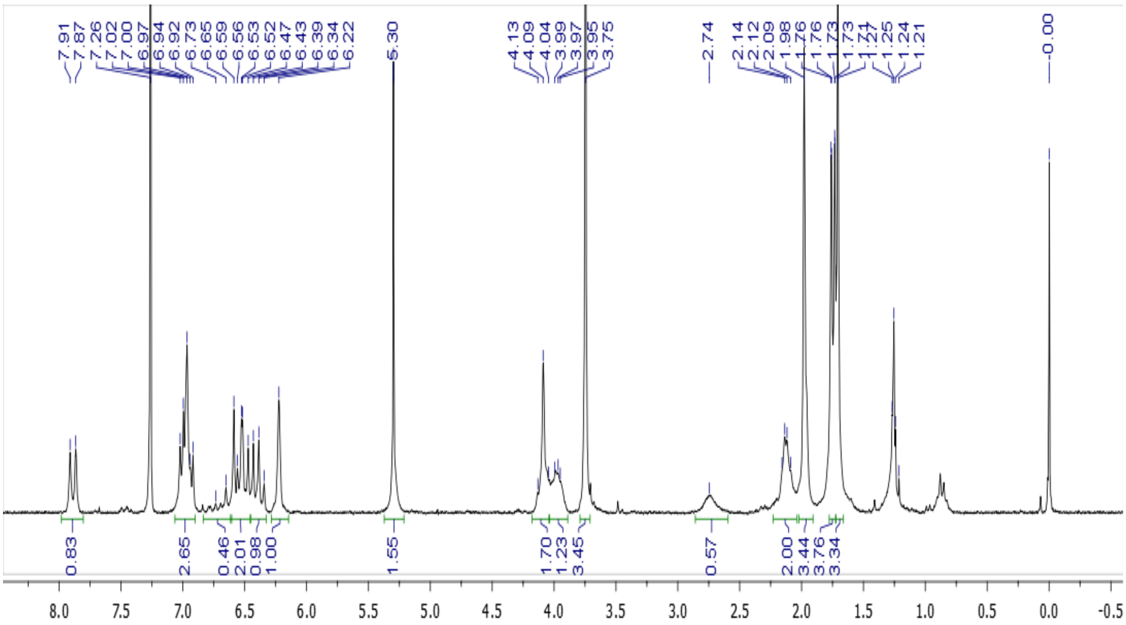

Figure S34: ^{1}H NMR spectrum of **9** (250 MHz, $CDCl_3$)

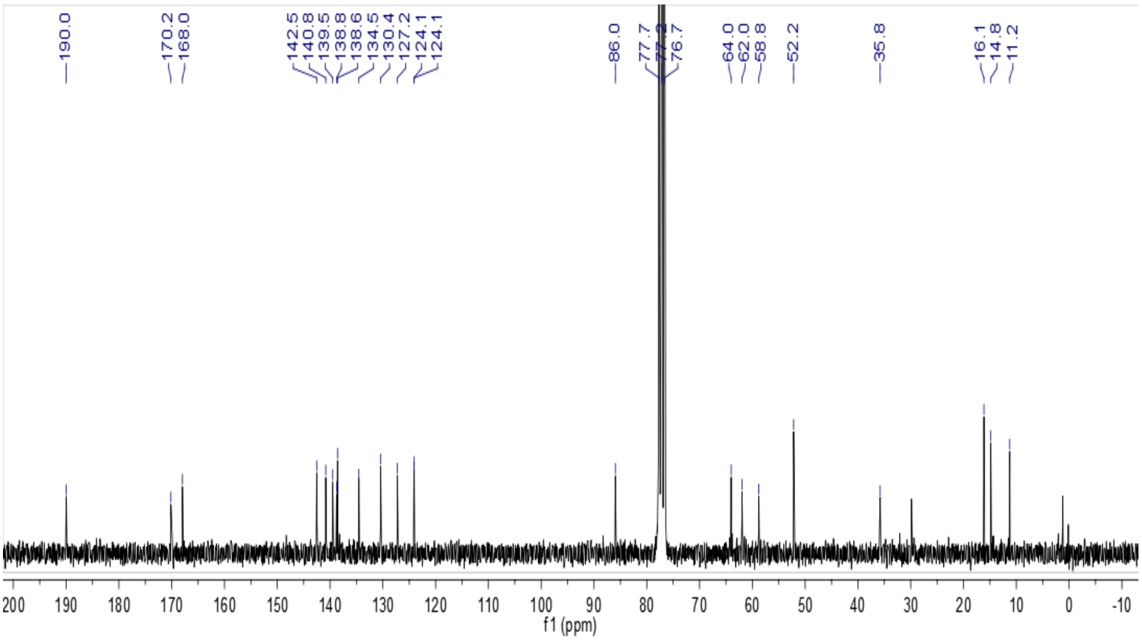

Figure S352: ^{13}C NMR spectrum of **9** (63 MHz, $CDCl_3$)

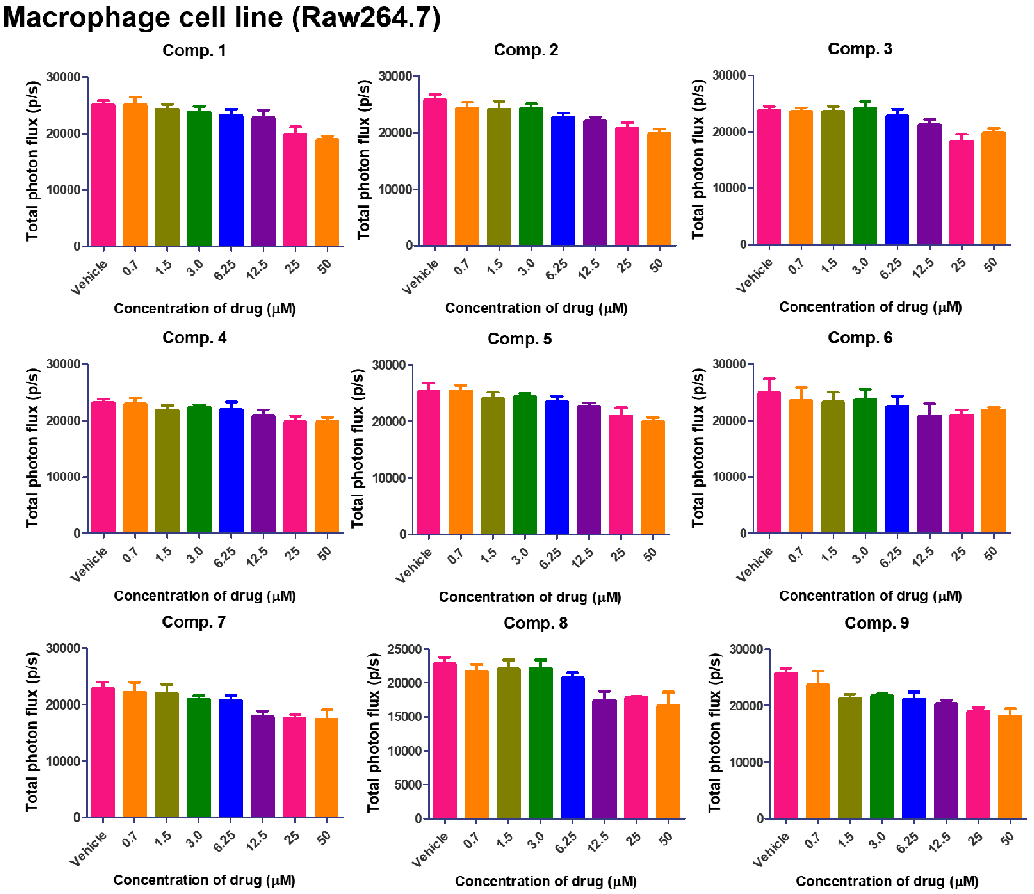

Figure S36: Effect on cell viability of compounds **1-9**. Cell proliferation in macrophage cell line (RAW264.7) with or without compounds **1-9**

Table S1: NMR spectroscopic data (600/ 150 MHz, $CDCl_3$) for **2** (δ in ppm)

Position	δ_H (multiplicity, *J* in Hz)	δ_C type	^1H-^1H COSY	HMBC	NOESY
1	1.75 (dd, 7.2, 1.3)	16.2	2	2, 3	2
2	7.00 (qd, 7.2, 0.6)	140.8		1, 4, 20,	1
3		130.4			
4	6.24 (br s)	128.3		2, 6, 22	6
5		138.4			4, 7
6	6.64 (d, 15.1)	143.0	7	4, 5, 8, 22	
7	6.95 (dd, 15.1, 11.7)	123.6	6. 8	5, 8	6, 10, 22
8	6.58 (dd, 11.7, 11.1)	139.8	7, 9	6	9
9	6.40 (dd, 11.6, 11.1)	124.1	8, 10	8, 10, 11	8, 23
10	7.97 (d, 11.6)	139.8		8, 12, 23	7, 13
11		134.8			
12		197.4			
13	4.38 (br s)	57.3		12, 14, 15, 17	10
14	4.29 (d, 0.6)	85.8		12, 13, 17, 19	15, 18, 19
15		94.5			
15-OH	5.00 (s)			15, 18	14
16-NH	6.18 (br s)			13, 14	18
17		170.4			
18a	2.28 (ddd, 12.7, 6.5, 3.6)	37.6	19	15, 19	19a, 19b
18b	2.45 (dt, 12.7, 8.8)				19a, 20b
19a	4.05 (td, 8.8, 6.5)	68.7	18		18a, 18b
19b	4.14 (td, 8.8, 3.6)				18a, 18b
20		167.7			
21	3.75 (s)	52.1		20	
22	1.79 (d, 1.0)	14.7		4, 5, 6	7
23	1.96 (d, 0.5)	11.5		10, 11, 12	9

Table S2: NMR spectroscopic data (600/ 150 MHz, $CDCl_3$) for **3** (δ in ppm)

Position	δ_H (multiplicity, *J* in Hz)	δ_C type	^1H-^1H COSY	HMBC	NOESY
1	1.72 (dd, 7.2, 1.4)	16.1	2	2, 3	
2	7.06 (qd, 7.2, 1.0)	141.2	1	1, 20	
3		130.0			
4	6.10 (br s)	126.5		5, 6	22
5		136.5			7
6	6.45 (d, 15.3)	136.8	7	4, 7, 8	8, 22
7	6.51 (dd, 15.3, 10.4)	129.9	8	6, 7, 9	5, 9
8	6.78 (dd, 14.6, 10.4)	143.9	7	6, 9	6, 10
9	6.68 (dd, 14.6, 11.5)	128.6	10	7, 8	7, 23
10	7.44 (d, 11.5)	145.5	9	8, 12, 23	8, 13
11		134.6			
12		197.1			
13	4.33 (br s)	56.8		12, 14, 15, 17	10
14	4.25 (d, 0.7)	85.8		12, 17	
15		94.6			
15-OH	4.97 (s)			18	
16-NH	6.06 (br s)			13, 14	
17		170.6			
18a	2.27 (m)	37.6			
18b	2.43 (dt, 12.7, 8.8)		15, 19		
19a	4.03 (td, 8.8, 6.4)	68.7	18		
19b	4.13 (td, 8.8, 3.8)				
20		167.6			
21	3.76 (s)	52.1	20	20	
22	2.03 (d, 1.3)	20.0	4, 6	4, 5, 6	4, 6
23	1.95 (d, 0.8)	11.7	10, 11, 12	10, 11, 12	

Table S33: Energy minimization of13*S**, 14*S**, 15*S** furanopyrrolidone (Case I)

Boltzmann distribution of energy minimized conformer

Conformer	Calculated Energy (G) (atomic units)	Relative Energy (kcal/mol)	Boltzmann Weights (%)
1	-667.003592	0.000000	100.0000000

Optimized Z-Matrixes of major conformer in the chloroform (Á)

Atom	X	Y	Z
C	0.575535	-0.27888	-0.81329
C	1.187996	0.581904	0.303179
C	2.658926	0.378873	-0.04979
C	2.622235	-1.14676	-0.42724
O	1.219585	-1.52219	-0.67733
N	0.404355	1.790049	0.107233
C	-0.87279	1.498722	-0.31686
C	-0.93418	-0.01566	-0.7487
O	-1.81002	2.27877	-0.37972
C	-1.77924	-0.83803	0.230537
O	-1.43985	-0.99298	1.398942
C	-3.04988	-1.42713	-0.31006
H	-1.39949	-0.05824	-1.73506
H	0.883657	0.180559	-1.76543
O	1.047982	0.076322	1.613862
H	3.324256	0.570891	0.793426
H	2.95968	0.994863	-0.90046
H	2.976344	-1.78101	0.38632
H	3.200926	-1.35103	-1.33319
H	0.57318	2.655287	0.608035
H	-3.57905	-1.97704	0.468577
H	-2.8194	-2.09128	-1.15164
H	-3.68544	-0.62535	-0.70523
H	0.141537	-0.28571	1.731312

Table S44: Energy minimization of three conformers of 13*S**, 14*S**, 15*R** furanopyrrolidone (Case II)

Boltzmann distribution of energy minimized conformers

Conformer	Calculated Energy (G) (atomic units)	Relative Energy (kcal/mol)	Boltzmann Weights (%)
1	-667.003592	0.000000	99.192498858
2	-667.179546	0.831450	0.262591423
3	-667.179709	0.729166	0.544909718

Optimized Z-Matrixes of major conformer in the chloroform (Á)

Atom	X	Y	Z
C	0.821479	0.41333	0.661622
C	0.352946	1.43944	-0.37649
C	-0.99732	1.289777	-0.51756
C	-1.62012	0.24795	0.28116
O	-0.38174	-0.55029	0.805513
N	-0.27072	-1.70384	-0.03182
C	-1.2717	-1.67858	-1.0766
C	-2.38771	-0.78473	-0.54617
O	2.156194	-0.30078	0.404171
C	2.813925	-0.66861	1.36594
O	2.597701	-0.53965	-1.0141
C	0.952435	0.954375	1.606446
H	1.042716	2.275028	-0.95023
H	-2.47618	0.776687	1.274291
O	-0.52239	-0.87777	1.838373
H	-1.53389	1.914234	-1.10825
H	-0.83201	-1.27625	-1.99853
H	-1.58499	-2.70884	-1.25706
H	-2.98839	-0.32397	-1.33457
H	-3.05037	-1.33745	0.125481
H	1.816033	-1.08292	-1.55551
H	3.526121	-1.11225	-1.02281
H	2.740525	0.420408	-1.52126
H	-2.02199	1.487703	1.750454

Table S55: Energy minimization of 13*S**, 14*R**, 15*R** furanopyrrolidone (Case III)

Boltzmann distribution of energy minimized conformer

Conformer	Calculated Energy (G) (atomic units)	Relative Energy (kcal/mol)	Boltzmann Weights (%)
1	-667.004109	0.000000	100.0000000

Optimized Z-Matrixes of major conformer in the chloroform (Á)

Atom	X	Y	Z
C	-1.30483	-1.55619	0.533442
C	-0.49225	-0.59233	-0.11771
C	-1.37184	0.655913	-0.05403
C	-2.65462	0.104863	-0.67293
O	-2.60626	-1.37232	-0.12517
N	-0.4849	1.627164	-0.62694
C	0.813419	1.356964	-0.22908
C	0.847015	-0.09292	0.388661
O	-0.3572	-0.88122	-1.16922
C	-1.61378	1.0593	1.304153
O	1.765264	2.108727	-0.3479
C	2.082853	-0.87964	-0.05908
H	3.371839	-0.56447	0.651071
H	2.002831	-1.70868	-0.95213
O	0.864595	0.040153	1.47627
H	-2.60537	0.118005	-1.76421
H	-3.54554	0.643624	-0.34553
H	-2.6929	-2.09794	-0.93967
H	-3.37141	-1.58115	0.624439
H	-0.75082	2.589296	-0.80333
H	-1.92362	0.286247	1.80263
H	3.563662	0.512883	0.592292
H	4.197447	-1.12435	0.20964
H	3.278609	-0.81725	1.714225

Table S66: Energy minimization of 13*S**, 14*R**, 15*S** furanopyrrolidone (Case IV)

Boltzmann distribution of energy minimized conformer

Conformer	Calculated Energy (G) (atomic units)	Relative Energy (kcal/mol)	Boltzmann Weights (%)
1	-667.033953	0.000000	100.0000000

Optimized Z-Matrixes of major conformer in the chloroform (Á)

Atom	X	Y	Z
C	-0.46787	-0.3386	0.885062
C	-1.15225	-0.55566	-0.49834
C	-2.63316	-0.32727	-0.20011
C	-2.58132	0.724072	0.907894
O	-1.37815	0.436927	1.663633
N	-0.56502	0.526408	-1.28698
C	0.528804	1.13535	-0.7574
C	0.827742	0.437601	0.588412
O	2.020104	-0.4938	0.360249
C	3.37486	-0.00786	0.785625
O	1.844381	-1.58301	-0.17816
C	1.048898	1.185025	1.352643
H	1.176202	2.047524	-1.25803
H	-0.28172	-1.29229	1.386545
O	-0.93518	-1.8161	-1.07959
H	-3.05944	-1.2663	0.163607
H	-3.19668	-0.00075	-1.0781
H	-3.42616	0.671247	1.598064
H	-2.51877	1.740793	0.50098
H	-0.89627	0.764072	-2.21444
H	3.568737	0.971271	0.331696
H	4.148478	-0.72016	0.496685
H	3.387437	0.139667	1.872182
H	0.021129	-2.00318	-1.01219

Table S7: Optical rotation calculation of *R*-solaniol

Boltzmann distribution of energy minimized conformers

Conformer	Calculated Energy (G) (atomic units)	Relative Energy (kcal/mol)	Boltzmann Weights (%)
1	-1032.926729	0.000000	0.000000000
2	-1032.934535	-4.898339	5.879042322
3	-1032.926582	0.092244	0.000000000
4	-1032.923683	1.911394	0.000000000
5	-1032.935154	-5.286768	94.036363748
6	-1032.933588	-4.304088	0.084593930
7	-1032.926293	0.273594	0.000000000
8	-1032.925225	0.943774	0.000000000
9	-1032.925172	0.977032	0.000000000

Specific rotation of *R*-solaniol [deg × dm^{-1} × $(g/100\ mL)^{-1}$] :+ **6.754**

Optimized Z-matrixes of *R*-solaniol conformers in the MeOH (Á)

Atom	Conformer 1			Conformer 2			Conformer 3		
	X	Y	Z	X	Y	Z	X	Y	Z
C	-3.31292	0.879007	0.188727	-3.32512	0.570182	0.198108	3.295143	0.952867	0.341526
C	-3.17878	-0.45869	0.018631	-3.08618	-0.75294	0.032286	3.230319	-0.39534	0.221385
C	-1.83248	-1.05249	-0.24804	-1.6969	-1.26342	-0.18078	1.912829	-1.071	0.012359
C	-0.6901	-0.15827	-0.24402	-0.61998	-0.2931	-0.20344	0.740803	-0.22871	-0.12766
C	-0.85406	1.233331	-0.03301	-0.88241	1.086569	-0.02384	0.839809	1.181739	-0.0303
C	-2.1866	1.785646	0.174875	-2.25381	1.546107	0.179165	2.132844	1.804576	0.222834
C	0.596813	-0.66708	-0.46066	0.698058	-0.71806	-0.39762	-0.51418	-0.80962	-0.3543
C	1.740895	0.181719	-0.45961	1.775914	0.203003	-0.42323	-1.68692	-0.01084	-0.50805
C	1.57651	1.538838	-0.23537	1.519286	1.553839	-0.23098	-1.58329	1.368618	-0.41338
C	0.264551	2.062444	-0.03114	0.177007	1.994461	-0.0376	-0.30518	1.960539	-0.17251
C	2.709916	2.525016	-0.19596	2.619811	2.581546	-0.23958	-2.74302	2.314266	-0.56052
C	3.086472	-0.45483	-0.71445	3.175367	-0.31371	-0.67689	-2.98604	-0.73501	-0.76638
C	3.813082	-0.93636	0.556795	3.915184	-0.83936	0.570798	-3.65592	-1.29912	0.504431
C	5.128935	-1.62577	0.214158	5.404901	-1.00383	0.307866	-4.93269	-2.05038	0.169136
O	-4.29165	-1.20177	0.006984	-3.99196	-1.72633	0.034578	4.36014	-1.09337	0.388164
C	-4.31612	-2.59591	0.397861	-5.372	-1.37642	0.232021	4.598045	-2.37046	-0.25191
O	-2.36072	3.014446	0.355257	-2.51205	2.761419	0.336907	2.247998	3.050003	0.318081
O	-1.71977	-2.26922	-0.48428	-1.50435	-2.48071	-0.32894	1.843155	-2.31355	-0.01465
O	0.1509	3.38454	0.161687	-0.03821	3.308952	0.126431	-0.25058	3.297606	-0.08922
O	0.795854	-1.97784	-0.67878	0.988734	-2.02476	-0.56032	-0.65852	-2.13822	-0.4419
O	2.97922	-1.78599	1.358994	3.403748	-2.1075	1.014965	-4.01651	-0.25167	1.420493
H	-4.29419	1.304938	0.356196	-4.32127	0.9599	0.35243	4.249568	1.432731	0.518151
H	2.701985	3.06894	0.751419	2.260987	3.536767	0.133631	-2.76663	3.01358	0.277497
H	3.681585	2.054615	-0.31021	3.464773	2.267138	0.373228	-2.62647	2.918307	-1.46507
H	2.591612	3.273873	-0.98352	2.994157	2.739606	-1.2558	-3.69762	1.801237	-0.60308
H	3.741093	0.241891	-1.23748	3.776097	0.48434	-1.11086	-2.78923	-1.57592	-1.43405
H	2.953928	-1.31113	-1.37927	3.141972	-1.11809	-1.41624	-3.69691	-0.08283	-1.27204
H	4.015843	-0.07359	1.196925	3.777044	-0.11865	1.386203	-2.95537	-1.97812	1.000675
H	5.798411	-0.94601	-0.31889	5.870969	-0.038	0.100635	-4.72202	-2.8814	-0.50637
H	5.628934	-1.95466	1.126713	5.891892	-1.43804	1.183102	-5.38723	-2.45056	1.076911
H	4.953855	-2.50029	-0.41865	5.575729	-1.66563	-0.54504	-5.6532	-1.38592	-0.31467
H	-3.66266	-2.77103	1.249975	-5.71289	-0.71232	-0.56358	4.198681	-2.37632	-1.26413
H	-4.02739	-3.23252	-0.4334	-5.5075	-0.90126	1.204706	4.16146	-3.1779	0.328585
H	-5.35047	-2.77996	0.678558	-5.91746	-2.31443	0.195171	5.681292	-2.4621	-0.27967
H	-0.82622	3.558879	0.280327	-1.02507	3.410802	0.24477	0.708802	3.520081	0.084488
H	-0.1035	-2.40627	-0.66504	0.122988	-2.51742	-0.50829	0.244638	-2.53053	-0.29708
H	2.489749	-2.38074	0.774732	2.545549	-2.27255	0.599082	-3.21053	0.136502	1.781947

Table S7: (continued)

Optimized Z-matrixes of *R*-solaniol conformers in the MeOH (Á)

Atom	Conformer 4			Conformer 5			Conformer 6		
	X	Y	Z	X	Y	Z	X	Y	Z
C	3.184822	1.009027	0.37541	3.309853	0.622734	0.2154	3.180808	0.761913	0.270625
C	3.125176	-0.33454	0.211863	3.12467	-0.71348	0.086098	3.062468	-0.57445	0.080441
C	1.810658	-1.00261	-0.03225	1.761928	-1.2863	-0.14299	1.734228	-1.19867	-0.2078
C	0.63621	-0.1569	-0.1556	0.650168	-0.36272	-0.21516	0.580824	-0.32491	-0.2683
C	0.729716	1.247444	-0.01046	0.857972	1.033086	-0.0786	0.719585	1.070947	-0.06853
C	2.021468	1.862109	0.274004	2.203266	1.554641	0.138147	2.033158	1.644169	0.205751
C	-0.61424	-0.73247	-0.40971	-0.64772	-0.84519	-0.42463	-0.68752	-0.85645	-0.52458
C	-1.78546	0.059695	-0.54459	-1.76472	0.038215	-0.50143	-1.84479	-0.02802	-0.58904
C	-1.68709	1.438543	-0.41019	-1.55749	1.402865	-0.36265	-1.701	1.340434	-0.41129
C	-0.41754	2.030186	-0.13687	-0.23224	1.89527	-0.15566	-0.40794	1.883836	-0.14256
C	-2.87494	2.351462	-0.56665	-2.65091	2.432856	-0.43117	-2.83172	2.327399	-0.49094
C	-3.09712	-0.61997	-0.88447	-3.12069	-0.58254	-0.73551	-3.16492	-0.70343	-0.88616
C	-3.83026	-1.38847	0.246392	-3.77631	-1.15831	0.537406	-3.91225	-1.30217	0.329442
C	-3.89724	-0.64078	1.573735	-5.11641	-1.80195	0.226392	-4.43305	-0.273	1.318884
O	4.254053	-1.03534	0.366379	4.068661	-1.64933	0.140624	4.047741	-1.46715	0.118681
C	4.497255	-2.29897	-0.29826	5.427441	-1.23803	0.36175	5.380736	-1.0037	0.38941
O	2.135189	3.102757	0.411322	2.409276	2.785373	0.256736	2.177313	2.876097	0.382711
O	1.740598	-2.24272	-0.10186	1.624016	-2.51547	-0.2618	1.656783	-2.42603	-0.38365
O	-0.35616	3.364044	-0.00944	-0.08009	3.222962	-0.03857	-0.32302	3.210809	0.034354
O	-0.74376	-2.06667	-0.54098	-0.88367	-2.15651	-0.56665	-0.8492	-2.17665	-0.73026
O	-3.30749	-2.71067	0.442645	-4.02218	-0.13291	1.514442	-3.09669	-2.21914	1.073023
H	4.135918	1.485734	0.576418	4.28673	1.054964	0.379853	4.131947	1.231404	0.477807
H	-2.64184	3.352121	-0.21363	-2.52256	3.063299	-1.31582	-2.97198	2.823641	0.47292
H	-3.16507	2.429124	-1.6191	-3.64081	1.99047	-0.45771	-2.59386	3.113116	-1.21133
H	-3.74059	1.985781	-0.01616	-2.59551	3.097919	0.432876	-3.773	1.869306	-0.77592
H	-2.92833	-1.32938	-1.69901	-3.00965	-1.40321	-1.44686	-2.98268	-1.51474	-1.59392
H	-3.78896	0.12738	-1.26857	-3.80431	0.138779	-1.18119	-3.84537	-0.01391	-1.38431
H	-4.85082	-1.54458	-0.11395	-3.1064	-1.90747	0.971124	-4.76778	-1.85011	-0.08581
H	-4.40765	0.31883	1.461492	-4.99378	-2.61232	-0.49445	-5.14958	0.397853	0.841415
H	-4.4547	-1.23588	2.298968	-5.56042	-2.21271	1.13464	-4.94037	-0.78071	2.141574
H	-2.89937	-0.45551	1.978095	-5.80703	-1.0667	-0.19449	-3.61784	0.321029	1.737253
H	4.108253	-2.28305	-1.31433	5.518537	-0.72882	1.322447	5.426534	-0.53172	1.372052
H	4.054298	-3.11788	0.261026	5.762975	-0.58434	-0.44476	5.70302	-0.30207	-0.38143
H	5.580573	-2.39084	-0.31651	6.01106	-2.15356	0.367633	6.006038	-1.89109	0.371807
H	0.60229	3.576549	0.180952	0.898123	3.372575	0.10048	0.642254	3.400649	0.207912
H	0.164072	-2.45687	-0.3955	-0.00224	-2.61224	-0.4888	0.055521	-2.58922	-0.64892
H	-2.35605	-2.69524	0.261114	-3.17568	0.18357	1.852489	-2.47789	-2.6503	0.467856

Table S7: (continued)

Optimized Z-matrixes of *R*-solaniol conformers in the MeOH (Á)

Atom	Conformer 7			Conformer 8			Conformer 9		
	X	Y	Z	X	Y	Z	X	Y	Z
C	3.309198	0.934309	0.342013	-3.317015	0.915052	0.213364	-3.293073	0.934601	0.36112
C	3.232128	-0.41285	0.222158	-3.2164	-0.428597	0.072633	-3.214228	-0.411415	0.229159
C	1.907961	-1.075568	0.016278	-1.890204	-1.057344	-0.21134	-1.890607	-1.068841	0.005268
C	0.743431	-0.222554	-0.118742	-0.727226	-0.190387	-0.240902	-0.728246	-0.211484	-0.131583
C	0.854059	1.186927	-0.023051	-0.854121	1.209207	-0.06291	-0.841375	1.196386	-0.024057
C	2.154045	1.796435	0.225323	-2.171412	1.795104	0.153386	-2.14106	1.800253	0.242308
C	-0.517915	-0.79303	-0.34245	0.54406	-0.736365	-0.466052	0.532673	-0.77788	-0.365045
C	-1.678657	0.017364	-0.488463	1.706052	0.085892	-0.489626	1.69197	0.036087	-0.509295
C	-1.566035	1.395486	-0.400351	1.576638	1.456937	-0.327374	1.572264	1.415666	-0.433558
C	-0.284871	1.979559	-0.165096	0.281485	2.017895	-0.112984	0.29305	1.994198	-0.17457
C	-2.753689	2.307661	-0.555523	2.760981	2.385946	-0.39038	2.750612	2.335189	-0.622239
C	-2.99177	-0.678162	-0.755373	3.036387	-0.586835	-0.727025	3.008897	-0.658774	-0.757233
C	-3.657992	-1.279488	0.489998	3.584162	-1.376437	0.484552	3.618742	-1.345082	0.487511
C	-4.895989	-2.089925	0.126552	4.005249	-0.498352	1.649408	4.090107	-0.374079	1.555325
O	4.355883	-1.122038	0.386695	-4.344734	-1.147924	0.101161	-4.33469	-1.124067	0.397443
C	4.582137	-2.396558	-0.262199	-4.387686	-2.526833	0.541361	-4.562901	-2.397101	-0.253864
O	2.284312	3.04101	0.318595	-2.318419	3.032145	0.301529	-2.272945	3.043364	0.348501
O	1.827284	-2.317732	-0.012642	-1.81132	-2.279228	-0.433928	-1.80663	-2.309855	-0.036751
O	-0.206032	3.316593	-0.084974	0.184534	3.347413	0.038412	0.210002	3.32973	-0.079488
O	-0.674842	-2.120767	-0.434382	0.711596	-2.04879	-0.677274	0.692003	-2.103859	-0.471078
O	-3.993349	-0.184522	1.361689	4.751247	-2.114958	0.086576	4.769653	-2.111239	0.094808
H	4.268032	1.406251	0.516036	-4.28455	1.367871	0.389915	-4.251327	1.402855	0.547649
H	-2.522161	3.308358	-0.199873	2.455143	3.415791	-0.231087	2.435869	3.374648	-0.613759
H	-3.041645	2.389395	-1.608685	3.506695	2.130624	0.363926	3.490595	2.199097	0.168927
H	-3.610786	1.92558	-0.004065	3.251479	2.323717	-1.365073	3.251998	2.136745	-1.571825
H	-2.817595	-1.494912	-1.458108	3.788455	0.139294	-1.033297	3.744381	0.036823	-1.159462
H	-3.696378	0.006996	-1.226875	2.917429	-1.296231	-1.550463	2.850137	-1.43472	-1.510968
H	-2.937141	-1.927635	0.998537	2.816727	-2.079508	0.820968	2.872625	-2.019844	0.916862
H	-4.628972	-2.941443	-0.503786	3.166065	0.099223	2.0095	3.264475	0.245283	1.9098
H	-5.377579	-2.478121	1.027394	4.3607	-1.116295	2.475747	4.492902	-0.922431	2.408585
H	-5.618716	-1.47094	-0.41086	4.812707	0.175019	1.351683	4.874572	0.278181	1.16404
H	4.181417	-2.392249	-1.273974	-3.719284	-2.684652	1.385407	-4.111775	-3.205294	0.314361
H	4.139332	-3.204314	0.313144	-4.128977	-3.197667	-0.272637	-4.171996	-2.387687	-1.269399
H	5.66453	-2.497543	-0.292311	-5.418819	-2.680821	0.850603	-5.645159	-2.501483	-0.27353
H	0.757754	3.522332	0.088242	-0.789073	3.535729	0.173464	-0.753159	3.530218	0.104871
H	0.224839	-2.521284	-0.293114	-0.194714	-2.457671	-0.647468	-0.205846	-2.508326	-0.329414
H	-4.347232	-0.548456	2.182014	4.4911	-2.772993	-0.56966	4.481608	-2.823018	-0.48969

Table S8: Optical rotation calculation of *S*-solaniol

Boltzmann distribution of energy minimized conformers

Conformer	Calculated Energy (G) (atomic units)	Relative Energy (kcal/mol)	Boltzmann Weights (%)
1	-1032.926729	0.000000	0.000000000
2	-1032.934535	-4.898339	5.879042322
3	-1032.926582	0.092244	0.000000000
4	-1032.923683	1.911394	0.000000000
5	-1032.935154	-5.286768	94.036363748
6	-1032.933588	-4.304088	0.084593930
7	-1032.926293	0.273594	0.000000000
8	-1032.925172	0.977032	0.000000000

Specific rotation of *S*-solaniol [deg × dm^{-1} × (g/100 mL)$^{-1}$] :**- 6.754**

Optimized Z-matrixes of *S*-solaniol conformers in the MeOH (Á)

Atom	Conformer 1			Conformer 2			Conformer 3		
	X	Y	Z	X	Y	Z	X	Y	Z
C	3.31288	0.87905	0.188742	3.325123	0.570182	0.198108	-3.295144	0.952867	0.341525
C	3.178752	-0.458654	0.01866	3.086183	-0.752941	0.032286	-3.230319	-0.395344	0.221386
C	1.832413	-1.052489	-0.247898	1.696902	-1.263416	-0.180778	-1.91283	-1.071001	0.012361
C	0.690039	-0.158253	-0.243877	0.619975	-0.293103	-0.203436	-0.740804	-0.228708	-0.12766
C	0.854007	1.233347	-0.032879	0.882406	1.086569	-0.023843	-0.83981	1.181739	-0.030299
C	2.186549	1.785651	0.175009	2.253814	1.546107	0.179165	-2.132845	1.804576	0.222833
C	-0.596858	-0.667063	-0.460528	-0.698058	-0.718058	-0.397616	0.514183	-0.809623	-0.354299
C	-1.74094	0.181732	-0.4596	-1.775914	0.203003	-0.423227	1.686919	-0.010839	-0.508047
C	-1.576561	1.538867	-0.235463	-1.519286	1.553839	-0.230983	1.583288	1.368617	-0.413378
C	-0.264607	2.062468	-0.031067	-0.177007	1.994461	-0.037596	0.305182	1.960538	-0.172506
C	-2.709949	2.525104	-0.196506	-2.619811	2.581546	-0.239576	2.743021	2.314266	-0.560515
C	-3.086474	-0.454837	-0.714496	-3.175367	-0.313706	-0.676887	2.986034	-0.735013	-0.766374
C	-3.812989	-0.936441	0.556804	-3.915184	-0.839355	0.570798	3.655921	-1.299122	0.504431
C	-5.128775	-1.626011	0.214238	-5.404901	-1.003834	0.307866	4.932694	-2.050376	0.169133
O	4.291633	-1.20168	0.006896	3.991962	-1.726327	0.034578	-4.360141	-1.093365	0.388164
C	4.316259	-2.595949	0.397276	5.371996	-1.376418	0.232021	-4.598044	-2.370456	-0.251909
O	2.36065	3.014452	0.355467	2.512054	2.761419	0.336908	-2.248	3.050003	0.31808
O	1.719697	-2.269214	-0.484053	1.504346	-2.480708	-0.328935	-1.843156	-2.31355	-0.014642
O	-0.150943	3.384561	0.161797	0.038213	3.308952	0.126431	0.250581	3.297606	-0.089222
O	-0.795924	-1.977851	-0.678491	-0.988734	-2.024761	-0.560316	0.658522	-2.138217	-0.441891
O	-2.979028	-1.785984	1.358986	-3.403748	-2.107502	1.014965	4.016512	-0.251672	1.42049
H	4.294151	1.305001	0.356128	4.321268	0.9599	0.35243	-4.249569	1.432732	0.518149
H	-3.681603	2.054697	-0.310823	-3.464773	2.267138	0.373228	2.626468	2.918308	-1.465063
H	-2.591437	3.273683	-0.984313	-2.994157	2.739606	-1.255796	3.69762	1.801236	-0.603082
H	-2.702184	3.069376	0.750662	-2.260987	3.536767	0.13363	2.766629	3.013578	0.277499
H	-2.953954	-1.311105	-1.379358	-3.141972	-1.118088	-1.416235	3.696908	-0.08283	-1.272042
H	-3.74118	0.241882	-1.237412	-3.776097	0.48434	-1.110861	2.789223	-1.575924	-1.434047
H	-4.015802	-0.073654	1.196898	-3.777044	-0.118653	1.386203	2.955368	-1.978119	1.000677
H	-4.953586	-2.500592	-0.418451	-5.575729	-1.665627	-0.545044	5.653196	-1.385922	-0.314675
H	-5.628771	-1.954842	1.126818	-5.891892	-1.438042	1.183102	5.387229	-2.450559	1.076907
H	-5.798318	-0.946382	-0.318897	-5.870969	-0.037996	0.100635	4.722022	-2.881396	-0.506369
H	4.027517	-3.232289	-0.434189	5.5075	-0.901257	1.204706	-4.16146	-3.177896	0.328582
H	3.662892	-2.771443	1.249387	5.712888	-0.712324	-0.563579	-4.198677	-2.37632	-1.264136
H	5.350661	-2.779982	0.677807	5.917463	-2.314426	0.195171	-5.681291	-2.4621	-0.279674
H	0.826176	3.558876	0.28048	1.025065	3.410802	0.24477	-0.708804	3.520079	0.084486
H	0.103413	-2.406264	-0.664722	-0.122988	-2.517422	-0.508289	-0.244637	-2.530527	-0.297076
H	-2.48915	-2.380362	0.774683	-2.545549	-2.272553	0.599082	3.210539	0.136506	1.781944

Table S8: (continued)

Optimized Z-matrixes of *S*-solaniol conformers in the MeOH (Ǻ)

Atom	Conformer 4			Conformer 5			Conformer 6		
	X	Y	Z	X	Y	Z	X	Y	Z
C	-3.184822	1.009027	0.37541	-3.30985	0.622734	0.2154	-3.180781	0.761901	0.270675
C	-3.125176	-0.334542	0.211863	-3.12467	-0.71348	0.086098	-3.062447	-0.574456	0.080456
C	-1.810658	-1.002609	-0.032252	-1.76193	-1.2863	-0.14299	-1.73423	-1.198657	-0.207865
C	-0.63621	-0.1569	-0.155595	-0.65017	-0.36272	-0.21516	-0.580817	-0.3249	-0.268333
C	-0.729716	1.247444	-0.010461	-0.85797	1.033086	-0.0786	-0.719567	1.070955	-0.068533
C	-2.021468	1.862109	0.274004	-2.20327	1.554641	0.138147	-2.033135	1.644165	0.205781
C	0.614235	-0.732469	-0.409714	0.647715	-0.84519	-0.42463	0.687528	-0.856435	-0.524637
C	1.785458	0.059695	-0.544589	1.76472	0.038215	-0.50143	1.844804	-0.028004	-0.589001
C	1.687086	1.438543	-0.410192	1.557488	1.402865	-0.36265	1.701033	1.340443	-0.411213
C	0.417537	2.030186	-0.136871	0.232238	1.89527	-0.15566	0.407961	1.88385	-0.142555
C	2.874938	2.351462	-0.566645	2.650907	2.432856	-0.43117	2.831768	2.327387	-0.490874
C	3.09712	-0.619968	-0.884474	3.120694	-0.58254	-0.73551	3.164903	-0.703458	-0.886146
C	3.830264	-1.388471	0.246392	3.776312	-1.15831	0.537406	3.912126	-1.302338	0.32947
C	3.897243	-0.640776	1.573735	5.116414	-1.80195	0.226392	4.433293	-0.27321	1.318764
O	-4.254053	-1.035338	0.366379	-4.06866	-1.64933	0.140624	-4.047722	-1.467144	0.118718
C	-4.497255	-2.298973	-0.29826	-5.42744	-1.23803	0.36175	-5.380686	-1.00366	0.389482
O	-2.135189	3.102757	0.411322	-2.40928	2.785373	0.256736	-2.177314	2.876096	0.382695
O	-1.740598	-2.242723	-0.101855	-1.62402	-2.51547	-0.2618	-1.656809	-2.426015	-0.383788
O	0.356157	3.364044	-0.009439	0.08009	3.222962	-0.03857	0.323029	3.210833	0.034283
O	0.743764	-2.066674	-0.54098	0.883673	-2.15651	-0.56665	0.849205	-2.176619	-0.730419
O	3.307492	-2.71067	0.442645	4.02218	-0.13291	1.514442	3.096313	-2.218851	1.073278
H	-4.135918	1.485734	0.576418	-4.28673	1.054964	0.379853	-4.131915	1.231388	0.4779
H	3.740591	1.985781	-0.016156	3.640812	1.990471	-0.45771	2.593945	3.113047	-1.211348
H	2.641836	3.352121	-0.213633	2.595514	3.097919	0.432876	3.773045	1.869248	-0.775798
H	3.165069	2.429124	-1.6191	2.522554	3.063299	-1.31582	2.971992	2.823718	0.472941
H	3.788956	0.12738	-1.268565	3.804305	0.138779	-1.18119	3.845459	-0.014008	-1.384233
H	2.928327	-1.329377	-1.699014	3.00965	-1.40321	-1.44686	2.982598	-1.514708	-1.593965
H	4.850821	-1.544583	-0.113953	3.106401	-1.90747	0.971124	4.76748	-1.850568	-0.085794
H	2.899368	-0.455513	1.978095	5.807029	-1.0667	-0.19449	3.618272	0.321099	1.73711
H	4.4547	-1.235876	2.298968	5.560416	-2.21271	1.13464	4.940519	-0.780961	2.141488
H	4.407645	0.31883	1.461492	4.993775	-2.61232	-0.49445	5.150002	0.397355	0.841165
H	-4.054298	-3.117881	0.261026	-5.76298	-0.58434	-0.44476	-5.702961	-0.301975	-0.381315
H	-4.108253	-2.283051	-1.314326	-5.51854	-0.72882	1.322447	-5.426464	-0.531726	1.372147
H	-5.580573	-2.390838	-0.316508	-6.01106	-2.15356	0.367633	-6.00602	-1.891031	0.371842
H	-0.60229	3.576549	0.180952	-0.89812	3.372575	0.10048	-0.642245	3.400708	0.207811
H	-0.164072	-2.456869	-0.395503	0.002235	-2.61224	-0.4888	-0.055534	-2.589173	-0.649056
H	2.356048	-2.695237	0.261114	3.175677	0.18357	1.852489	2.477702	-2.650311	0.46813

Table S8: (continued)

Optimized Z-matrixes of *S*-solaniol conformers in the MeOH (Á)

Atom	Conformer 7			Conformer 8		
	X	Y	Z	X	Y	Z
C	-3.309198	0.934309	0.342012	3.293073	0.934601	0.36112
C	-3.232128	-0.41285	0.222158	3.214228	-0.411415	0.229159
C	-1.907961	-1.075568	0.016278	1.890607	-1.068841	0.005268
C	-0.743431	-0.222554	-0.118742	0.728246	-0.211484	-0.131584
C	-0.854059	1.186927	-0.023051	0.841375	1.196386	-0.024057
C	-2.154045	1.796435	0.225323	2.14106	1.800253	0.242308
C	0.517915	-0.79303	-0.34245	-0.532673	-0.77788	-0.365045
C	1.678657	0.017364	-0.488463	-1.69197	0.036087	-0.509295
C	1.566035	1.395486	-0.400351	-1.572264	1.415666	-0.433558
C	0.284871	1.979559	-0.165096	-0.29305	1.994198	-0.17457
C	2.753689	2.307661	-0.555523	-2.750612	2.335189	-0.622239
C	2.99177	-0.678162	-0.755373	-3.008897	-0.658774	-0.757233
C	3.657992	-1.279488	0.489998	-3.618742	-1.345082	0.487511
C	4.895989	-2.089925	0.126552	-4.090107	-0.374079	1.555325
O	-4.355883	-1.122038	0.386695	4.33469	-1.124067	0.397443
C	-4.582137	-2.396558	-0.262199	4.562901	-2.397101	-0.253864
O	-2.284312	3.04101	0.318595	2.272945	3.043364	0.348501
O	-1.827284	-2.317732	-0.012642	1.80663	-2.309855	-0.036751
O	0.206032	3.316593	-0.084974	-0.210002	3.32973	-0.079488
O	0.674842	-2.120767	-0.434382	-0.692003	-2.103859	-0.471078
O	3.993349	-0.184522	1.361689	-4.769653	-2.111239	0.094808
H	-4.268032	1.406251	0.516036	4.251327	1.402856	0.547649
H	3.041645	2.389395	-1.608685	-3.490595	2.199098	0.168928
H	3.610786	1.92558	-0.004065	-3.251999	2.136745	-1.571825
H	2.522161	3.308358	-0.199873	-2.435869	3.374648	-0.613759
H	3.696378	0.006996	-1.226875	-2.850137	-1.43472	-1.510968
H	2.817595	-1.494912	-1.458108	-3.744381	0.036823	-1.159462
H	2.937141	-1.927635	0.998537	-2.872625	-2.019844	0.916862
H	5.618716	-1.47094	-0.41086	-4.874572	0.278181	1.16404
H	5.377579	-2.478121	1.027394	-4.492902	-0.922431	2.408585
H	4.628972	-2.941443	-0.503786	-3.264474	0.245283	1.9098
H	-4.139332	-3.204314	0.313144	4.171996	-2.387687	-1.269399
H	-4.181417	-2.392249	-1.273974	4.111775	-3.205294	0.314361
H	-5.66453	-2.497543	-0.292311	5.645159	-2.501483	-0.27353
H	-0.757754	3.522332	0.088242	0.753159	3.530218	0.104871
H	-0.224839	-2.521284	-0.293114	0.205846	-2.508326	-0.329414
H	4.347232	-0.548456	2.182014	-4.481608	-2.823018	-0.48969
